# Role of eosinophil counts in mediating the association between asthma and colon cancer

**DOI:** 10.1002/clt2.70012

**Published:** 2024-12-10

**Authors:** Zhi‐Qing Zhan, Ze‐Min Huang, Zhi‐Xin Xie, Hao‐Bin Zhou, Yu‐Hua Luo, Pei‐Zhen Chen, Tian‐Ye Luo, Baoqing Sun, Zhangkai J. Cheng

**Affiliations:** ^1^ Department of Clinical Laboratory National Center for Respiratory Medicine National Clinical Research Center for Respiratory Disease State Key Laboratory of Respiratory Disease Guangzhou Institute of Respiratory Health The First Affiliated Hospital of Guangzhou Medical University Guangzhou Medical University Guangzhou China; ^2^ Division of Gastroenterology and Hepatology Shanghai Institute of Digestive Disease NHC Key Laboratory of Digestive Diseases State Key Laboratory for Oncogenes and Related Genes Renji Hospital School of Medicine Shanghai Jiao Tong University Shanghai China; ^3^ Department of Clinical Medicine Guangzhou Medical University Guangzhou China

**Keywords:** asthma, colon cancer, immune, Mendelian randomization, PPP1R14A

## Abstract

**Background:**

Epidemiological findings regarding the association between asthma and the risk of colon cancer (CC) are inconsistent. The causality and potential molecular mechanisms underlying asthma, eosinophil count, and CC remain unknown.

**Methods:**

We performed Mendelian randomization (MR) analysis to investigate the causality between asthma and CC and attempted to demonstrate that asthma indirectly affects CC mediated by eosinophil count through mediation analysis. Sensitivity analyses and multivariable MR were performed to test the robustness of our findings. Multiple bioinformatics tools were applied to further investigate the underlying mechanisms related to eosinophils that contribute to the pathogenesis of both asthma and CC.

**Results:**

MR with mediation analyses suggested that eosinophil count may play a role in the mechanism through which asthma reduces the risk of CC. Our bioinformatic analyses identified PPP1R14A as a potential therapeutic target and an eosinophil‐associated biomarker for CC patients. Higher expression of PPP1R14A may be associated with a poorer prognosis in CC patients. Additionally, the lysosome pathway emerges as a shared eosinophil‐related pathway in both asthma and CC.

**Conclusions:**

Eosinophils may contribute to a lower risk of CC in patients with asthma. PPP1R14A is a potential therapeutic target and biomarker for CC.

## 1 INTRODUCTION

Each year, more than 2 million individuals worldwide suffer from colon cancer (CC), making it the second leading cause of cancer deaths and the third most prevalent cancer.^1^ There has been considerable interest and debate regarding the potential link between asthma and CC. Two conflicting theories have been proposed regarding this matter. The immune surveillance hypothesis suggests that asthma, an allergy‐related condition, may indicate a heightened immune system that can identify and eliminate cancer cells, potentially reducing the risk of developing cancer.^2^ Conversely, the antigen stimulation hypothesis proposes that chronic immune system activation resulting from allergy‐related conditions could increase the occurrence of pro‐oncogenic mutations. This ongoing inflammation, tissue damage, and subsequent repair processes may contribute to the development of cancer.^3^ It is essential to clarify the association and causality between asthma and CC because it may support primary interventions and clinical prevention strategies for CC. However, epidemiological findings regarding the association between asthma and CC are inconsistent. Large‐scale record linkage studies conducted in Europe comparing the incidence of colorectal cancer in asthma patients to that of the general population have produced diverse results. These studies have reported a decreased risk,^4^ an increased risk,^5^ or no significant association^6^ between asthma and the risk of colorectal cancer. Furthermore, Prizment et al. found that individuals with two or more allergy‐related conditions had a lower risk of developing colorectal cancer.^7^ More importantly, previous observational studies cannot fully establish a causal relationship between asthma and CC due to confounding factors and reverse causal effects. For instance, patients with asthma might adopt a different lifestyle compared with healthy individuals. They may reduce their smoking habits, avoid certain foods, or choose occupations that have lower levels of chemical hazard exposure. These confounding factors could potentially impact the risk of CC. Thus, the causality between asthma and CC remains unknown.

Mendelian randomization (MR) is a reliable method that employs genetic variants to evaluate the potential causal relationship between a risk factor and an outcome. By leveraging genotypes that are not influenced by disease, MR helps overcome bias arising from confounding or reverse causation.^8^ Mediation analysis decomposes the effects of an exposure on an outcome to understand the underlying mechanisms.^8^ Eosinophils, as a type of innate immune leukocytes, are often associated with type 2 immune responses such as asthma. Loktionov et al. found that eosinophils in the gut of healthy individuals help maintain the mucosal barrier and contribute to gut‐associated immunity.^9^ Lima‐Matos et al. found that elevated blood eosinophil levels are linked to asthma severity and poor symptom control.^10^ Interestingly, previous studies have suggested that higher blood eosinophil counts may be associated with a reduced risk of developing^10^ and a better prognosis in vitro and in vivo.^11^, ^12^ These findings suggest that eosinophil counts is a part of the mechanism for explaining the causality between asthma and CC risk.

**FIGURE 1:**
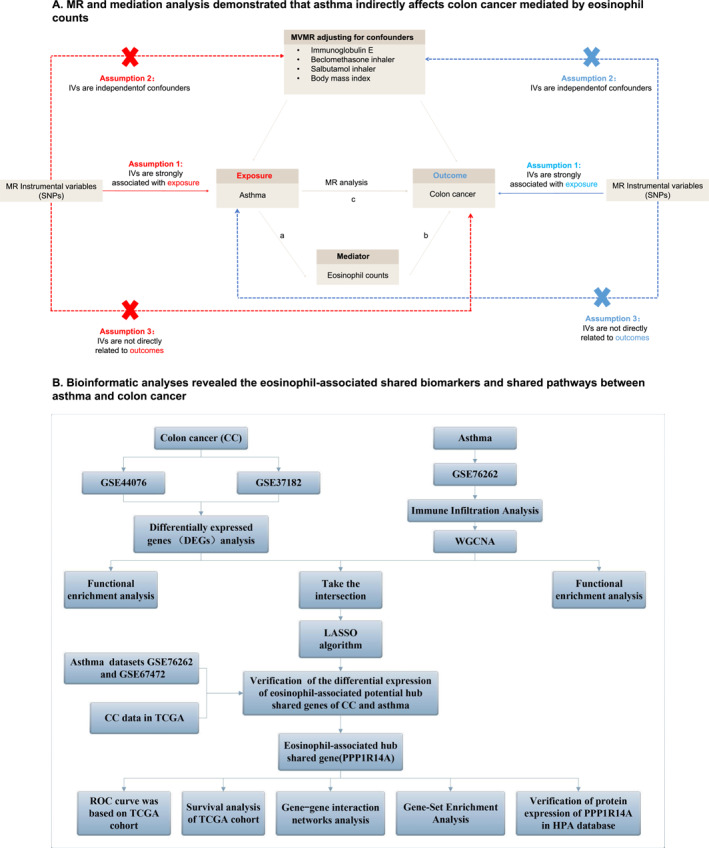
Workflow of this study design. (A) Mendelian randomization revealed the causality between rheumatoid arthritis and colorectal cancer. (B) Bioinformatic analyses revealed eosinophil‐associated shared biomarkers and shared pathways between asthma and colon cancer.

In the present study, we aim to address the following issues: (i) Is there a causal association between asthma and the risk of CC? (ii) If such an association exists, does asthma indirectly affect CC mediated by eosinophil counts? (iii) If so, what are the underlying mechanisms related to eosinophils that contribute to the pathogenesis of both asthma and CC? We performed an MR analysis to investigate the causality between asthma and CC and attempted to demonstrate that asthma indirectly affects CC mediated by eosinophils through mediation analysis. In addition, exploring the underlying eosinophil‐associated shared biomarkers and molecular mechanisms between asthma and CC is crucial as it may provide new insights into the relationship between innate immunity and cancer and potential therapeutic implications. Hence, we further applied multiple bioinformatics analyses and protein immunohistochemical staining to investigate the eosinophil‐associated shared biomarkers and shared pathways related to the pathogenesis of asthma and CC. Figure 1 depicts the study design flow chart.

## 2 MATERIAL AND METHODS

### 2.1 Mendelian randomization (MR) and mediation analysis

#### 2.1.1 Data source and selection of instrumental variables (IVs)

To explore the causal relationship between asthma, eosinophil counts, and CC. Genome‐wide association study (GWAS) data of the aforementioned diseases were obtained from the IEU Open GWAS Project. Detailed information on the GWAS data is shown in Supplementary Table 1. We chose IVs based on the following criteria: (1) strong association with exposures, (2) no association with confounding factors, and (3) no direct association with outcomes.^8^ To meet the first assumption, we chose SNPs that were associated with each trait at a significance threshold of *p* < 5 × 10^−8^. Only SNPs with a long physical distance (≥10,000 kb) and less possibility of linkage disequilibrium (*R* ^2^ < 0.001) were retained. We manually excluded IVs that were significantly associated (*p* < 5 × 10^−8^) with confounders.

#### 2.1.2 Statistical methods

We utilized 5 MR approaches, namely IVW, weighted median, MR Egger, simple mode, and weighted mode, to investigate the causal effects between asthma, eosinophil count, and CC.^8^ In addition, we conducted the MR‐Egger intercept test to assess the presence of directional pleiotropy in the genetic variants and examined heterogeneity using Cochran's *Q* test.^8^ To assess the potential mediating effect of eosinophil count in the association between asthma and CC, we conducted a mediation analysis. Mediation analysis was based on the following two steps: first, the impact of the exposure on the mediators was calculated, and second, the effect of the mediators on the outcome was determined.^8^ In our study, we first utilized UVMR to estimate the effect of asthma on the eosinophil count. Then, we employed multivariate MR (MVMR) to investigate the asthma‐adjusted effect of eosinophil count on CC. The mediation effect can be calculated using the following formula: $\beta_{\text{mediating}}=\beta_{xz}\times \beta_{zy}^{\prime }$,^13^ where $\beta_{xz}$ represents the effect of exposure on mediating calculated by UVMR and $\beta_{zy}^{\prime }$ represents the effect adjusted by the exposure of mediations on the outcome calculated by MVMR. To assess the reliability of the estimated mediating effect, we calculated the standard error using both the delta method and the bootstrap method.^14^ The proportion of the mediating effect was determined by calculating the ratio of the mediating effect size to the summary effect size.

In addition, we conducted MVMR analysis to explore the causality between asthma, eosinophil count, and CC after adjusting for potential confounders, including immunoglobulin E, Beclomethasone inhaler, Salbutamol inhaler, and body mass index. Details of the GWAS summary data of the potential confounders are presented in Supplementary Table S1. A significance level of *p* < 0.05 (two‐sided) was used to determine statistical significance. All the above processes were carried out in R 4.1.0 using the “TwoSampleMR” package and the “MVMR” package.

### 2.2 Bioinformatic analysis

#### 2.2.1 Data download and processing

The datasets GSE44076 and GSE37182 were obtained from the Gene Expression Omnibus (GEO) for preliminary gene differential analysis of CC. Data from The Cancer Genome Atlas (TCGA) database were used for further analysis. The GSE44076 dataset consists of 98 CC samples and 98 paired control samples, and the GSE37182 dataset consists of 82 pairs. To detect the differentially expressed genes (DEGs) between the CC group and the control group, the Dplyr and DESeq2 packages were used for paired difference analysis. Statistical screening standards were set as |logFC|≥1 and adjusted *p* < 0.05. The intersection of the results from the two datasets was considered as DEGs of CC. A volcano plot was generated using the ggplot2 package to visualize the results. For the analysis of asthma, 118 asthma samples and 21 control samples were downloaded from the GSE76262 dataset. Additionally, the GSE67472 dataset, which contains 62 asthma samples and 43 control samples, was used for validation.

#### 2.2.2 Immune infiltration analysis

CIBERSORT is a validated computational method that accurately estimates immune cell compositions in different cancer types using RNA sequencing data.^15^ In this study, we utilized the CIBERSORT algorithm to analyze immune cell infiltration characteristics in asthma using the GSE76262 dataset. Statistical tests were assessed using the Wilcoxon rank sum test. Additionally, given the widespread use of carcinoembryonic antigen (CEA) in the diagnosis and prognostic evaluation of CC, we employed the ssGSEA algorithm^16^ to investigate the relationship between CEA and immune cell infiltration in CC. The differences in immune infiltration between high and low CEA expression groups (median split) were analyzed using Welch's *t*‐test. RNA‐seq data for CC were sourced from the TCGA‐COAD cohort. The analysis was conducted using the R package GSVA [version 1.46.0], and the results were visualized using the ggplot2 package.

#### 2.2.3 Identification of eosinophil‐associated genes in asthma using weighted gene coexpression network analysis (WGCNA)

WGCNA is a powerful algorithm that analyzes gene expression patterns in multiple samples. It can cluster genes and construct modules based on similar expression patterns, while also examining the relationships between these modules and biological traits.^17^ To identify modules closely associated with eosinophils in the asthma dataset GSE76262, the WGCNA approach was employed. Initially, genes with a variance (median absolute deviation before 45%, MAD) greater than 0.01 were selected to construct co‐expression networks using the R package “WGCNA.” Outlier samples were removed using a CutHeight of 105. We selected a soft threshold of 4 meeting the criteria for scale‐free topology. To visualize the results, a hierarchical clustering dendrogram was generated with the criteria minModuleSize set to 50 and mergeCutHeight set to 0.1. By calculating the correlations between module eigengenes (ME) and eosinophil enrichment, modules that showed a positive correlation with eosinophil infiltration were identified. The genes within these modules, obtained through the aforementioned steps, were considered eosinophil‐associated genes in the context of asthma.

#### 2.2.4 Functional enrichment analysis

Gene Ontology (GO) and Kyoto Encyclopedia of Genes and Genomes (KEGG) enrichment analyses were conducted to explore the biological functions of the DEGs in CC and eosinophil‐associated genes in asthma. These analyses aimed to identify the functional annotations and pathways associated with these genes. The DAVID database was utilized for performing the enrichment analyses.

#### 2.2.5 Identification and verification of shared eosinophil‐associated hub genes

DEGs of CC intersected with eosinophil‐associated genes in asthma. The intersecting genes were identified as eosinophil‐associated shared genes of both CC and asthma. The least absolute shrinkage and selection operator (LASSO) Cox regression is a machine‐learning technique that minimizes classification errors to identify relevant variables in a model.^18^ In this context, LASSO was utilized to screen the potential eosinophil‐associated hub shared genes in CC and asthma. The LASSO algorithm was employed with the “glmnet” package. The asthma datasets GSE76262 and GSE67472, along with nonpaired and paired tissues from the CC data in the TCGA database, were utilized for verification of the shared eosinophil‐associated hub genes. The gene validated through this step was considered an eosinophil‐associated hub shared gene in CC and asthma and was utilized for the subsequent analyses.

#### 2.2.6 Identification of the eosinophil‐associated shared biomarker by receiver operating characteristic (ROC) analysis and prognostic analysis

To assess the diagnostic performance of the identified hub genes, ROC curves were employed. The “pROC” and “ggplot2” packages were utilized to generate these curves. The area under the curve (AUC) was calculated, which is a measure of diagnostic performance. A higher AUC indicates a better diagnostic performance for the hub genes in distinguishing between the disease group and control groups. We investigated the relationship between the expression of shared eosinophil‐associated hub genes and patient prognosis in CC. Relevant data for analysis were obtained from the TCGA database. KM grouping was performed using the minimum *p*‐value approach. Genes that showed significant results in both KM analysis and ROC analysis were considered eosinophil‐associated shared biomarkers in CC and asthma. The Wilcoxon rank sum test was applied to explore the correlation between the expression of the eosinophil‐associated shared biomarker and clinical features in CC.

#### 2.2.7 Gene‒gene interaction networks of the eosinophil‐associated shared biomarker

GeneMANIA database is a powerful tool that allows researchers to identify and predict proteins that exhibit similar functions and have the potential to interact with the key genes of interest. We utilized the GeneMANIA database to explore the interactions between the eosinophil‐associated shared biomarker and its associated genes. This analysis helped us investigate the biological mechanisms and functions in which these genes are involved.

#### 2.2.8 Identification of the shared pathways by gene set enrichment analysis (GSEA)

GSEA was employed to investigate the potential biological functions and pathways associated with hub genes exhibiting different expression levels in CC and asthma. The samples were divided into two groups based on the median level of gene expression. The asthma dataset GSE76262 and CC data in TCGA were applied to analysis. GSEA was then performed using the c2.cp.kegg.v2023.1.Hs.symbols.gmt gene set as a reference. This gene set contains annotated pathways from the KEGG database. Normalized enrichment score (|NES|) > 1, *q* value < 0.25 and *p* < 0.058 were considered filtering criteria.

#### 2.2.9 Verification of the expression of eosinophil‐associated shared biomarkers

The Human Protein Atlas (HPA) is a comprehensive database that contains abundant transcriptomic and proteomic data, offering valuable resources such as protein expression profiles, subcellular localization information, and immunohistochemistry images.^19^ Protein expression immunohistochemical (IHC) staining images of the eosinophil‐associated shared biomarker were obtained from the HPA database. These images offer visual evidence of the differential expression and localization of eosinophil‐associated shared biomarkers within CC and normal colon tissue.

## 3 RESULTS

### 3.1 Results of MR analysis and mediation analysis

**FIGURE 2:**
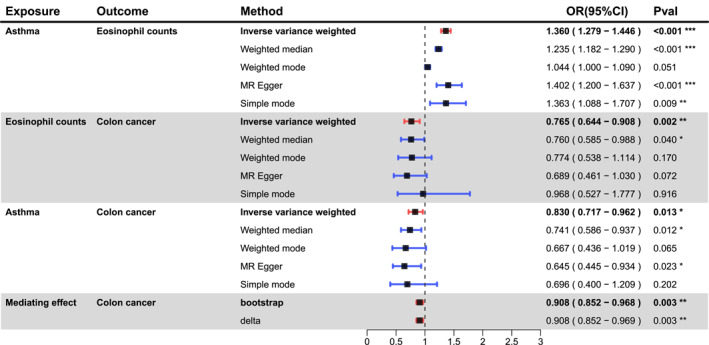
Forest plot showing the results of univariate MR analysis and mediation analysis.

Our UVMR analysis results showed that genetically predicted asthma was negatively associated with the risk of CC (OR = 0.830, 95% CI = 0.717–0.962, *p* = 0.013) but positively associated with eosinophil counts (OR = 1.360, 95% CI = 1.279–1.446, *p* = 1.09 × 10^−22^). Genetically predicted elevated eosinophil counts significantly decreased the risk of CC (OR = 0.765, 95% CI = 0.644–0.908, *p* = 0.002) (Figure 2 and Supplementary Table S2). The results of the mediation analysis showed that eosinophil counts mediate the relationship between asthma and CC. The proportion of asthma affecting CC via eosinophil counts accounted for 51.72% of the total mediating effect (Figure 2 and Supplementary Table S3). In sensitivity analyses, although heterogeneity was detected in some of our results based on Cochran's *Q* test, it did not invalidate the MR results, which might balance the pooled heterogeneity. The MR‐Egger intercept test found no evidence of unbalanced pleiotropy (Supplementary Table S2). In the MVMR analysis, after adjusting for potential confounders (i.e., immunoglobulin E, Beclomethasone inhaler, Salbutamol inhaler, and body mass index), the effect of eosinophil counts on CC was not substantially altered, which adds confidence to our finding that asthma indirectly affects CC mediated by eosinophils (Supplementary Table S4).

### 3.2 Results of bioinformatic analysis

#### 3.2.1 Results of DEGs in CC and immune infiltration analysis

**FIGURE 3:**
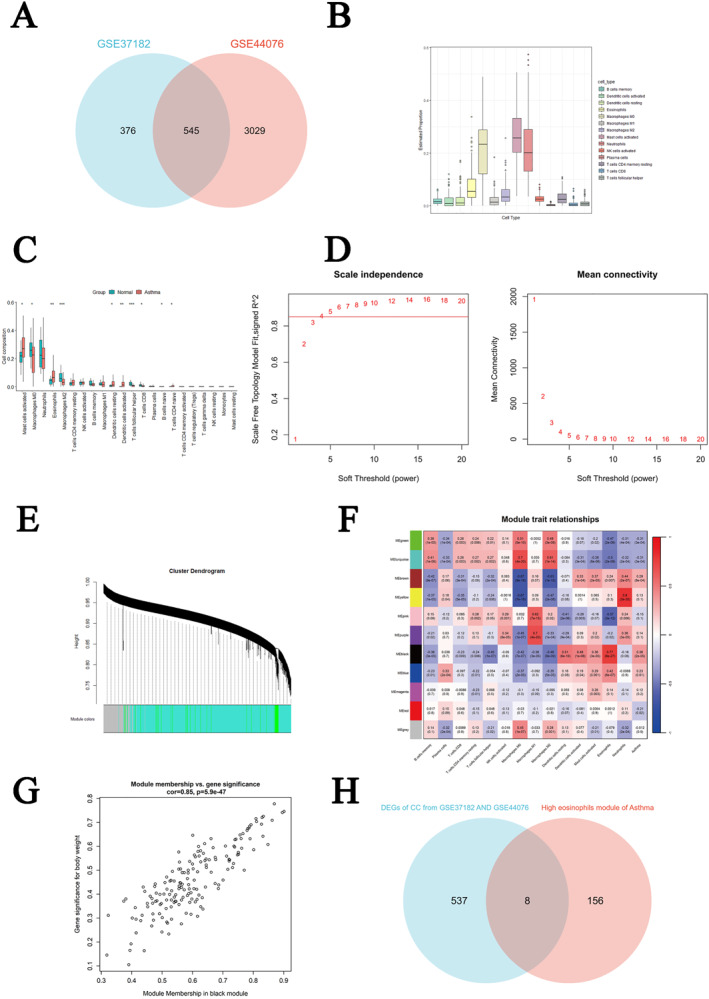
(A) The Venn diagram displays the overlap of DEGs in colon cancer obtained from the datasets GSE44076 and GSE37182. (B) The immune infiltration analysis revealed significant infiltration of 14 different types of immune cells. (C) A comparison of 22 different types of immune cell infiltration patterns between the asthma group and control group. (D) The optimal soft‐threshold power. (E) Cluster dendrogram of coexpressed genes in asthma. (F) Heatmap of module‐trait relationships in asthma. (G) Scatter plot of gene significance versus module membership in the black module. (H) Intersection of DEGs of CC and black module genes in asthma.

A total of 545 DEGs were identified by intersecting the CC datasets GSE44076 and GSE37182. Among these DEGs, 274 genes were upregulated, while 271 genes were downregulated (Figure 3A and Supplementary Figure S1). The CIBERSORT algorithm was applied to the asthma dataset GSE76262. Significant differences in the infiltration of 10 immune cell types were observed between the disease and control groups (Figure 3B–C).

#### 3.2.2 WGCNA identified 164 eosinophil‐related genes in asthma

WGCNA was applied to the asthma dataset GSE76262. We set CutHeight = 105 to eliminate outlier samples from the sample cluster tree, resulting in 129 remaining samples. Using *R* ^2^ = 0.85 as the scale‐free topology criterion, the optimal soft threshold was determined to be 4 using the pickSoftThreshold function (Figure 3D). Eleven modules were identified, with the black module exhibiting the strongest positive correlation with eosinophils (Cor = 0.77, *p* = 8 × 10^−27^) (Figure 3E,F). The results demonstrated a positive correlation between genes in the black module and eosinophils (Figure 3G). Finally, a total of 164 genes in the black module were identified as eosinophil‐related genes in asthma.

#### 3.2.3 Results of functional enrichment analysis

GO and KEGG analyses were performed on the DEGs of CC and the eosinophil‐related genes in asthma, respectively (Supplementary Tables S5 and S6). The enrichment results for both diseases are presented in Supplementary Figure S2. In terms of biological pathways, neutrophil chemotaxis was found to be significantly enriched in both diseases. Cytosol emerged as an important cellular component enriched in both diseases. Regarding molecular functions, protein binding was found to be predominantly activated.

#### 3.2.4 PPP1R14A was identified as an eosinophil‐associated hub shared gene

Eight eosinophil‐associated shared genes were obtained through the intersection of DEGs of CC and eosinophil‐related genes in asthma (Figure 3H). The LASSO algorithm was applied to further screen out eosinophil‐associated hub shared genes of asthma and CC. This screening process identified five genes (PPP1R14A, FNBP1, DRAM1, FSCN1, and MMP12) as potential shared eosinophil‐associated hub genes (Supplementary Figure S3). The asthma datasets GSE76262 and GSE67472 were employed to validate the differential expression of these genes. Through nonpair and pair verification analyses using TCGA data of CC, PPP1R14A was found to exhibit significant expression differences in both CC and asthma and was identified as an eosinophil‐associated hub shared gene (Supplementary Figure S4).

#### 3.2.5 PPP1R14A was identified as an eosinophil‐associated shared biomarker

**FIGURE 4:**
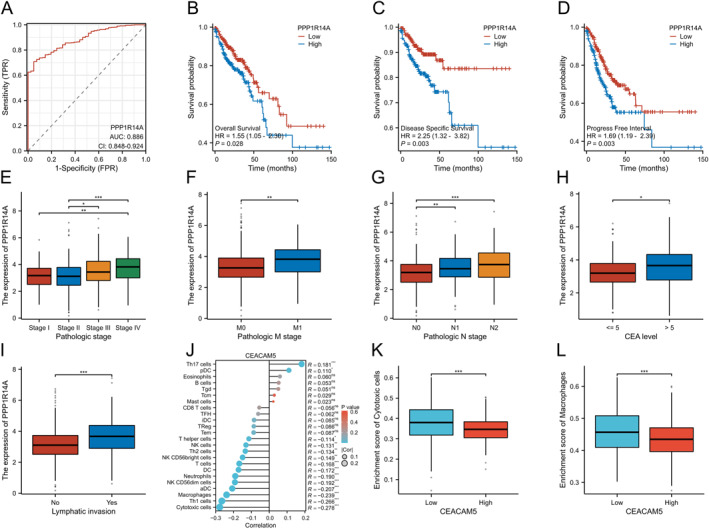
(A) The ROC curve showing the diagnostic accuracy of PPP1R14A in distinguishing between colon cancer and normal colon tissues. Kaplan‒Meier curve showing the association between PPP1R14A expression and the prognosis or pathological stages of patients with colon cancer: (B) overall survival; (C) disease‐specific survival; (D) progression‐free interval; (E) pathological stage; (F) pathological M stage; (G) pathological N stage; (H) CEA level; (I) lymphatic invasion status. The relationship between CEA expression and immune cell infiltration; (J) correlation analysis results with 24 immune cell types; (K) Relationship between high and low CEA expression groups and the enrichment score of cytotoxic cells; (L) Relationship between high and low CEA expression groups and the enrichment score of macrophages.

To evaluate the practicality of PPP1R14A in distinguishing between CC and normal tissue, diagnostic ROC curves were constructed. The AUC for PPP1R14A was 0.886, indicating its high accuracy in discriminating between CC and normal tissue (Figure 4A). PPP1R14A expression was observed to be downregulated in CC tissues. In the KM curve analysis, the hazard ratios (HRs) of PPP1R14A for disease‐specific survival (DSS), overall survival (OS), and progression‐free interval (PFI) were all found to be statistically significant (*p* < 0.05) and greater than 1, suggesting that lower PPP1R14A expression was associated with a better prognosis of CC (Figure 4B–D). Figure 4E–H illustrate a significant association between lower PPP1R14A expression and a lower pathological stage as well as a lower level of CEA (*p* < 0.05). In contrast, samples with lymphatic invasion exhibited higher levels of PPP1R14A expression (*p* < 0.05) (Figure 4I). The ssGSEA results indicated that CEA expression is negatively correlated with the immune infiltration of cytotoxic cells, Th1 cells, and macrophages (*p* < 0.001), while it is positively correlated with the infiltration of Th17 cells (*p* < 0.001). These findings were further corroborated by comparing immune infiltration results between the high and low CEA expression groups (Figure 4J–L).

#### 3.2.6 Biological processes associated with PPP1R14A and its related genes

**FIGURE 5:**
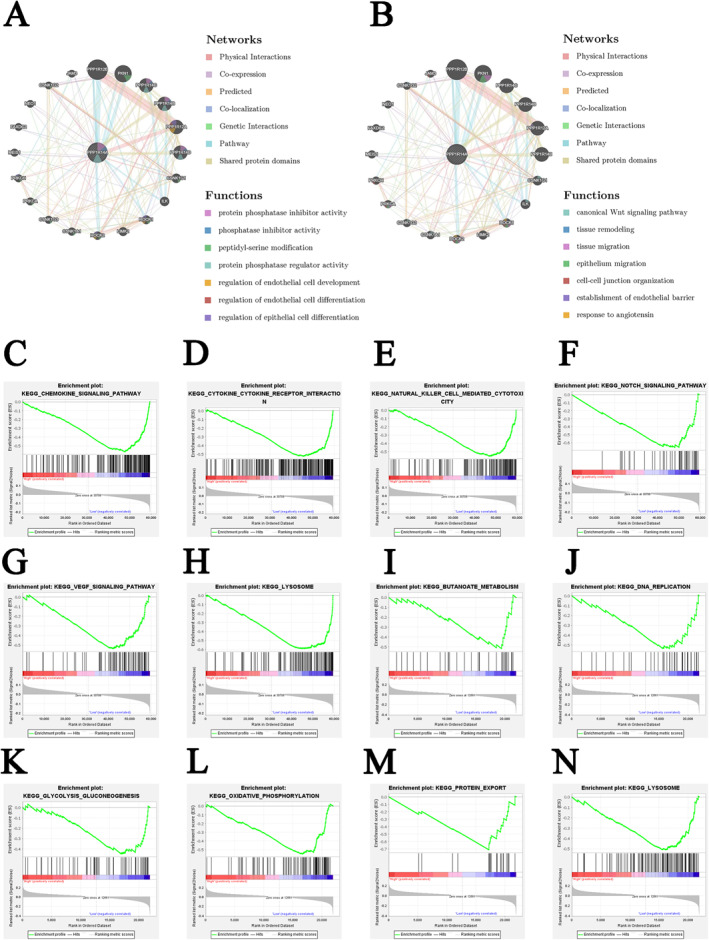
Biological processes associated with PPP1R14A and its related genes. (A, B) Results of GeneMANIA showing the interactions between PPP1R14A and its related genes; (C–H) the main signaling pathways that show significant enrichment in the low PPP1R14A expression group, based on colon cancer data obtained from TCGA; (I–N) the main signaling pathways that show significant enrichment in the low PPP1R14A expression group, based on asthma dataset GSE76262.

The results of GeneMANIA analysis illustrated the genes that exhibited significant interactions with PPP1R14A (Figure 5A,B). The biological functions of PPP1R14A and its related genes are primarily associated with protein phosphatase activity. They are also highly involved in the regulation of endothelial and epidermal development and differentiation, cell‒cell junctions, tissue remodeling and migration, the canonical Wnt signaling pathway, and the response to angiotensin.

#### 3.2.7 Eosinophil‐related shared pathways involving PPP1R14A

GSEA was utilized to explore the biological characteristics associated with PPP1R14A (Supplementary Tables S7 and S8). In CC, the PPP1R14A low expression group exhibited activation in various pathways, including the chemokine signaling pathway, Notch signaling pathway, natural killer cell‐mediated cytotoxicity, cytokine‒cytokine receptor interaction, vascular endothelial growth factor signaling pathway, and asthma (Figure 5C‐H). In asthma, oxidative phosphorylation, protein export, DNA replication, glycolysis gluconeogenesis, and butanoate metabolism were highly enriched in the PPP1R14A low expression group (Figure 5I–N). Additionally, the lysosome pathway was enriched in the PPP1R14A low expression groups in both CC and asthma (Figure 5H,N). Thus, the lysosome pathway was considered an eosinophil‐related shared pathway of asthma and CC.

#### 3.2.8 The protein IHC staining results of PPP1R14A

**FIGURE 6:**
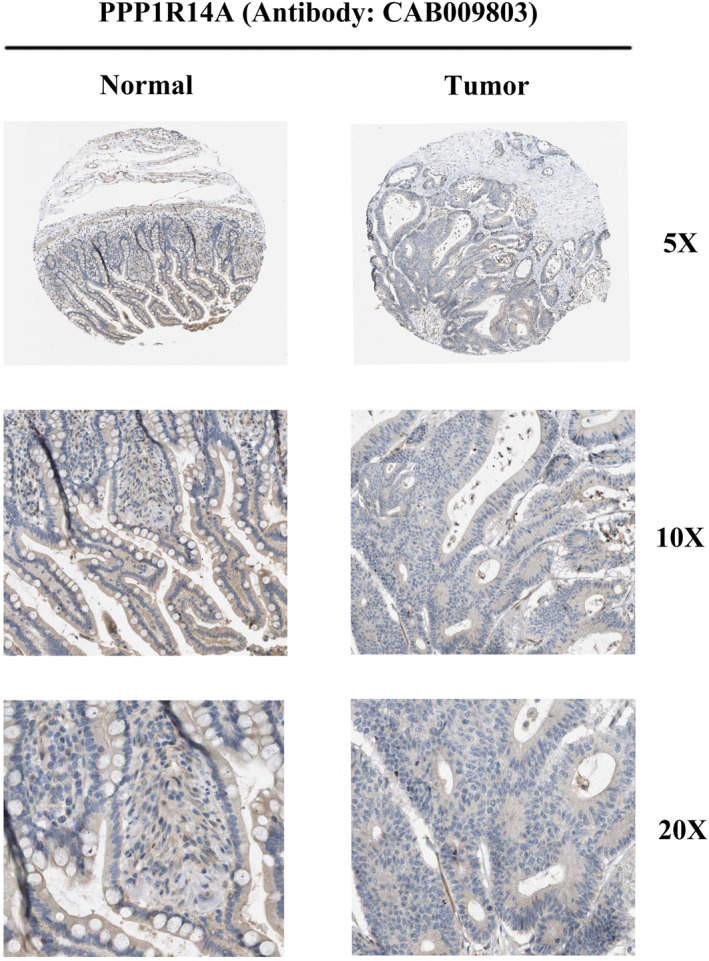
Protein expression of PPP1R14A in colon cancer and normal colon tissue (antibody CAB009803).

As discussed above, we further analyzed PPP1R14A expression at the protein level in normal colon and CC tissue using data available in the HPA repository. Antibody CAB009803 was used for IHC staining of PPP1R14A. The results obtained from IHC staining were consistent with the transcriptional level observed before, further validating the reliability of PPP1R14A that we found (Figure 6).

## 4 DISCUSSION

Research results thus far suggest a dual behavior of asthma in tumorigenesis. On the one hand, asthma, as an allergic disease, is considered a hyperreactive state of the immune system. This enhanced immune response improves the surveillance of cancerous cells, providing a protective effect against cancer development.^2^ On the contrary, the antigen stimulation hypothesis proposes that chronic immune system stimulation in an allergic state can lead to increased levels of random pro‐oncogenic mutations, repeated tissue inflammation, and damage. These factors may promote the development of cancer.^3^ Specifically, the association between asthma and cancer risk is particularly prominent in organs that directly interact with the environment, such as the respiratory and digestive systems.^20^ Previous studies have reported strong inverse associations between asthma and pancreatic cancer,^21^ glioma,^22^ and lymphoma.^23^ However, a positive association has been observed between asthma and lung cancer.^24^ However, previous studies examining the relationship between asthma and CC have yielded inconsistent findings.^4^, ^5^, ^6^, ^7^ Investigating the link between asthma and colon cancer is crucial as it can provide valuable insights into the underlying mechanisms of colon cancer and potentially lead to new therapeutic strategies.

Our MR study demonstrated that asthma indirectly reduces the risk of CC mediated by increased eosinophil counts. Further bioinformatic analysis revealed that PPP1R14A is an eosinophil‐associated shared biomarker in asthma and CC, which is the first discovery reported publicly. Previous cohort studies indicated that both asthma and elevated blood eosinophil counts were associated with a decreased risk of CC.^4^, ^25^, ^26^ Jacobs EJ et al. found that individuals who had both hay fever and asthma had a modestly lower risk of colorectal cancer mortality.^25^ Van Hulst G et al. showed that blood and lung eosinophilia is a hallmark of asthma.^27^ Circulating eosinophils can be recruited to the gut lining, exerting cytotoxic effects and potentially displaying antitumor activity on precancerous cells.^28^ Stimulated eosinophils have the ability to produce and rapidly release over 30 cytokines, primarily promoting type 2 immunity. They also release reactive nitric oxide, oxygen species, and mitochondrial DNA, which can have cytotoxic effects on tumor cells.^29^, ^30^ Eosinophils contribute to immune surveillance by synergizing with macrophages and releasing immunoregulatory cytokines, which enhance antitumor responses. On the other hand, depending on the danger signal, both cell types can also promote tumorigenesis.^31^ Furthermore, eosinophils can directly recognize CC cells and induce their death by releasing cytotoxic granzyme A.^26^, ^28^, ^32^

By using the LASSO algorithm, we identified five shared eosinophil‐associated hub genes (PPP1R14A, FNBP1, DRAM1, FSCN1, and MMP12) in asthma and CC. Through intersections with the validation dataset, we finally identified PPP1R14A as an eosinophil‐associated shared biomarker, which was found to be overexpressed in asthma and downregulated in CC compared to normal tissues. PPP1R14A, also known as 17 kDa PKC potentiated inhibitory protein of PP1 (CPI‐17), belongs to the protein phosphatase 1 (PP1) inhibitor family. PPP1R14A exhibits potent inhibitory activity, suppressing phosphorylation by more than 1000‐fold. This regulatory function allows PPP1R14A to control the phosphorylation state of PPP1CA substrates and regulate smooth muscle contraction.^33^ Previous studies have reported elevated expression of CPI‐17 protein in lung biopsies of asthma patients,^34^ while a downregulation of PPP1R14A expression has been observed in CC,^33^ which aligns with our current findings. Furthermore, the ROC curve indicates that PPP1R14A, with an AUC value of 0.886, achieved an outstanding level in the diagnostic test evaluation. This suggests that PPP1R14A is a potential valuable diagnostic biomarker for CC. We observed that PPP1R14A had lower expression in CC compared with normal colon tissue, indicating its potential role as a tumor suppressor gene. DNA methylation is a vital epigenetic modification that regulates gene expression while preserving the DNA sequence.^35^ Li et al. found that the expression of PPP1R14A in CC cell lines is controlled by methylation of the promoter region.^36^ Promoter region hypermethylation is a common mechanism associated with the silencing or inactivation of tumor suppressor genes in cancer cells,^35^ which may explain the downregulation of PPP1R14A in CC in our results. However, molecular biology‐based experiments are needed to confirm our findings. Furthermore, we observed a gradual increase in PPP1R14A expression with tumor progression in CC. Increased expression of PPP1R14A has been associated with a poorer prognosis for patients with CC. Interestingly, we found that the expression of PPP1R14A parallels that of CEA during tumor progression. Notably, CEA itself possesses potential immunoregulatory properties: its expression is negatively correlated with that of cytotoxic cells, Th1 cells, and macrophages. Consistent with our findings, Shao et al. demonstrated that the expression of CEA on intestinal epithelial cells is involved in the activation of regulatory T cell populations, exerting immunosuppressive effects within the tumor microenvironment.^37^ These findings suggest that PPP1R14A may play contrasting roles in the initiation and progression of cancers. Similarly, Wang et al. discovered that the role of PPP1R14A in bladder urothelial carcinoma and kidney renal papillary cell carcinoma differs between tumor and normal samples, which contrasts with its role in tumor cohorts.^33^ Experimental data have demonstrated genetic alterations in PPP1R14A in tumors. Although the frequency of these alterations may not be as high as anticipated, they still have a significant negative impact on the prognosis of OS, DFS, DSS, and PFS, which is consistent with the results of our survival analysis. To gain a comprehensive understanding of PPP1R14A and its associated molecules, we performed an analysis using GeneMANIA. The results demonstrated that PPP1R14A and its related genes are primarily associated with protein phosphatase activity and are also highly involved in the regulation of endothelial and epidermal development and differentiation, cell‒cell junctions, tissue remodeling and migration, the canonical Wnt signaling pathway, and the response to angiotensin. The canonical Wnt signaling pathway, also referred to as the Wnt/β‐catenin signaling pathway, is a well‐established driver of CC as well as one of the most prominent signaling pathways involved in CC pathogenesis.^38^ Thus, we speculate that the regulatory effects of PPP1R14A on the development of CC are likely to be associated with the canonical Wnt signaling pathway. However, further investigations are warranted to fully clarify the processes.

Several limitations should be acknowledged in this study. Firstly, to reduce heterogeneity, our MR analysis only included GWAS data from individuals of European ancestry. Consequently, the generalizability of our findings to other populations may be limited. Second, the limited availability of data on asthma prevented us from further exploring whether different phenotypes of asthma have varying impacts on CC. Third, this study may not have fully accounted for all potential confounding factors influencing the association between asthma, eosinophil count, and colon cancer risk. Unmeasured or residual confounding could potentially impact the validity of the results. Therefore, further large‐scale studies are required to validate and expand our findings in this context. Finally, although we propose several potential molecular mechanisms, our study lacks in vitro and in vivo experimental validation. Experimental validation is crucial for confirming the proposed mechanisms and providing direct evidence to support or refute our hypotheses. The absence of experimental validation may limit the robustness of our conclusions regarding the underlying molecular mechanisms. Future studies should focus on conducting experimental validation, expanding the sample size, and performing multi‐center data validation to enhance the robustness of our conclusions.

## 5 CONCLUSION

In conclusion, our study revealed that eosinophil count might be a part of the mechanism by which asthma reduces the risk of CC. In addition, we explored the molecular mechanism between asthma and CC for the first time and identified that PPP1R14A is a potential therapeutic target and an eosinophil‐associated biomarker for patients with CC. Higher expression of PPP1R14A might be associated with a worse prognosis for CC patients. Our findings enhance a better understanding of their shared underlying biology, which may provide an entry point for research into the pathogenesis and pharmacological mechanisms of action of those diseases as well as a basis for further translational studies, especially the role of eosinophils in reducing the risk of CC. Further randomized clinical trials and molecular biology‐based experiments are needed to confirm our findings.

## AUTHOR CONTRIBUTIONS

**Zhi‐Qing Zhan**: Conceptualization; methodology; formal analysis; writing—original draft. **Ze‐Min Huang**: Data curation; formal analysis; writing—original draft. **Zhi‐Xin Xie**: Software; data curation. **Hao‐Bin Zhou**: Software; data curation. **Yu‐Hua Luo**: Validation. **Pei‐Zhen Chen**: Visualization. **Tian‐Ye Luo**: Visualization. **Baoqing Sun**: Project administration; supervision. **Zhangkai J. Cheng**: Supervision; project administration.

## CONFLICT OF INTEREST STATEMENT

None.

## CONSENT FOR PUBLICATION

All authors have reviewed and approved the manuscript for submission to the journal.

## Supporting information

Supporting Information S1

Supporting Information S2

Figure S1

Figure S2

Figure S3

Figure S4

## Data Availability

The datasets generated and/or analyzed in this study can be found in the GEO database repository [https://www.ncbi.nlm.nih.gov/geo/] and MRC‐IEU [https://gwas.mrcieu.ac.uk/].
