# Supplementary material for: Role of eosinophil counts in mediating the association between asthma and colon cancer
Source: Clin Transl Allergy. 2024 Dec 10;14(12):e70012. doi: 10.1002/clt2.70012 (PMC11632118; doi:10.1002/clt2.70012)
Supplement: Supplementary file 1 — Supporting Information S1 [file CLT2-14-e70012-s006.docx]

**Supplementary Figure 1.** (A) Volcano plot presents DEGs of colon cancer (analysis based on the GSE44076 dataset); (B) Heatmap showing the expression levels of 20 DEGs between colon cancer and normal colon samples (analysis based on the GSE44076 dataset); (C) Volcano plot presents DEGs of colon cancer (analysis based on the GSE37182 dataset); (D) Heatmap showing the expression levels of 50 DEGs between colon cancer and normal colon samples (analysis based on the GSE37182 dataset); (E) Venn diagram showing the intersection of DEGs of colon cancer obtained from datasets GSE44076 and GSE37182.

**Supplementary Figure 2.** Results of the eosinophil-associated shared pathways in asthma and colon cancer based on GO and KEGG enrichment analysis. (A) The shared pathways in colon cancer; (B) the shared pathways in asthma.

**Supplementary Figure 3.** Coefficient distribution for the log(lambda) sequence in the LASSO regression algorithm.

**Supplementary Figure 4.** Validation of potential hub genes using the datasets GSE76262 and GSE67472 and colon cancer data in TCGA.
