# Supplementary material for: Role of eosinophil counts in mediating the association between asthma and colon cancer
Source: Clin Transl Allergy. 2024 Dec 10;14(12):e70012. doi: 10.1002/clt2.70012 (PMC11632118; doi:10.1002/clt2.70012)
Supplement: Supplementary file 2 — Supporting Information S2 [file CLT2-14-e70012-s005.docx]

**Supplementary Table 1**. Detailed information of the GWAS data for the MR study and potential confounders.

| **Dataset.id** | **Traits** | **ncases** | **ncontrol** | **Sample size** | **Year** | **Author** | **PMID** | **Population** |
| --- | --- | --- | --- | --- | --- | --- | --- | --- |
| ebi-a-GCST90014325 | Asthma | 56167 | 352255 | 408442 | 2021 | Valette K | 34103634 | European |
| ebi-a-GCST004606 | Eosinophil counts | NR | NR | 172275 | 2016 | Astle WJ | 27863252 | European |
| finn-b-C3_COLON | Malignant neoplasm of colon | 1803 | 216989 | 218792 | 2021 | NR | NR | European |
| prot-a-1456 | Immunoglobulin E | NR | NR | 3,301 | 2018 | Sun BB | 29875488 | European |
| ukb-a-124 | Treatment/medication code: becotide 50 inhaler | 2,063 | 335,096 | 337,159 | 2017 | Neale | NR | European |
| ukb-b-13148 | Treatment/medication code: ventolin 100micrograms inhaler | 13,244 | 449,689 | 462,933 | 2018 | Ben Elsworth | NR | European |
| ukb-b-2303 | Body mass index (BMI) | NR | NR | 454,884 | 2018 | Ben Elsworth | NR | European |

**Supplementary Table 2.** Results of univariate Mendelian randomization

| **Exposure** | **Outcome** | **Methods** | **nsnp** | **OR (95%CI)** | **P-value** | **P pleiotropy** | **P heterogeneity** |
| --- | --- | --- | --- | --- | --- | --- | --- |
| Asthma | Eosinophil counts | MR Egger | 78 | 1.40 (1.20-1.64) | 5.69E-05 | 0.674 | 4.19E-255 |
|  |  | Weighted median | 78 | 1.24 (1.18-1.29) | 2.78E-21 |  |  |
|  |  | Inverse variance weighted | 78 | 1.36 (1.28-1.45) | 1.09E-22 |  |  |
|  |  | Simple mode | 78 | 1.36 (1.09-1.71) | 0.009 |  |  |
|  |  | Weighted mode | 78 | 1.04 (1.00-1.09) | 0.051 |  |  |
| Eosinophil counts | Colon cancer | MR Egger | 160 | 0.69 (0.46-1.03) | 0.072 | 0.577 | 0.107 |
|  |  | Weighted median | 160 | 0.76 (0.59-0.99) | 0.040 |  |  |
|  |  | Inverse variance weighted | 160 | 0.76 (0.64-0.91) | 0.002 |  |  |
|  |  | Simple mode | 160 | 0.97 (0.53-1.78) | 0.916 |  |  |
|  |  | Weighted mode | 160 | 0.77 (0.54-1.11) | 0.170 |  |  |
| Asthma | Colon cancer | MR Egger | 76 | 0.64 (0.45-0.93) | 0.023 | 0.150 | 0.210 |
|  |  | Weighted median | 76 | 0.74 (0.59-0.94) | 0.012 |  |  |
|  |  | Inverse variance weighted | 76 | 0.83 (0.72-0.96) | 0.013 |  |  |
|  |  | Simple mode | 76 | 0.70 (0.40-1.21) | 0.202 |  |  |
|  |  | Weighted mode | 76 | 0.67 (0.44-1.02) | 0.065 |  |  |

**Supplementary Table 3.** Results of Mediation mendelian randomization analysis.

| **Exposure** | **Mediator** | **Outcome** | **Beta of mediating effect** | **Proportion of mediating effect** | **Method of calculating SE** | | **SE** | **P** |
| --- | --- | --- | --- | --- | --- | --- | --- | --- |
| Asthma | Eosinophil counts | Malignant neoplasm of colon | -0.0961 | 51.72% | bootstrap | 0.0324 | | 0.003 |
|  |  |  |  |  | delta | 0.0327 | | 0.0033 |

**Supplementary Table 4.** Multivariate mendelian randomization showed that the effect of eosinophil counts on colon cancer were not substantially altered after adjusting for potential confounders

| **The confounders being adjusted for** | **Exposure** | **Outcome** | **nsnp** | **Beta** | **SE** | **P-value** |
| --- | --- | --- | --- | --- | --- | --- |
| Immunoglobulin E | Eosinophil counts | Colon cancer | 147 | 0.297 | 0.100 | 0.003* |
|  | Asthma | Colon cancer | 45 | -0.055 | 0.103 | 0.593 |
|  | Immunoglobulin E | Colon cancer | 0 | 0.095 | 0.101 | 0.352 |
| Becotide inhaler | Eosinophil counts | Colon cancer | 144 | -0.280 | 0.100 | 0.005* |
|  | Asthma | Colon cancer | 45 | -0.185 | 0.153 | 0.226 |
|  | Becotide inhaler | Colon cancer | 0 | 20.618 | 14.802 | 0.164 |
| Ventolin inhaler | Eosinophil counts | Colon cancer | 138 | -0.280 | 0.103 | 0.007* |
|  | Asthma | Colon cancer | 46 | 0.200 | 0.282 | 0.478 |
|  | Ventolin inhaler | Colon cancer | 17 | -8.056 | 8.190 | 0.325 |
| Body mass index | Eosinophil counts | Colon cancer | 99 | -0.334 | 0.103 | 0.001* |
|  | Asthma | Colon cancer | 32 | 0.092 | 0.092 | 0.322 |
|  | Body mass index | Colon cancer | 289 | 0.057 | 0.122 | 0.637 |

**Supplementary Table 5.** Results of GO and KEGG analyses of the eosinophil-related genes in asthma.

| **Category** | **Term** | **Count** | **%** | **P-value** | **Genes** |
| --- | --- | --- | --- | --- | --- |
| CC | GO:0005886~plasma membrane | 60 | 40.816 | ＜0.001 | CLIC4, SIGLEC9, CSF1, SLC7A11, FLVCR1, FFAR3, VSTM1, ITGAV, CPNE2, CCR3, PPP1R16B, CISH, FNBP1, PDE4D, SYTL1, RHOF, ARL4C, DXO, SEMA7A, CALCRL, SUCNR1, TTYH2, CEBPE, ALOX15, CD1E, CD1C, CD1B, GPR82, LY9, CD1A, PIK3R6, IL1RL1, GNA15, APH1B, P2RY2, HRH4, CD300LB, S1PR1, FYN, CTNNAL1, P2RY10, MARCKSL1, TMEM120A, RFTN1, CTNS, PILRB, SVIP, XKR8, P2RX5, USH1G, CD209, GPR183, SLCO4C1, CD207, FCGR2B, SIGLEC8, CD24, ITM2A, GAPT, CRLF2 |
| BP | GO:0048006~antigen processing and presentation, endogenous lipid antigen via MHC class Ib | 4 | 2.721 | ＜0.001 | CD1E, CD1C, CD1B, CD1A |
| MF | GO:0030883~endogenous lipid antigen binding | 4 | 2.721 | ＜0.001 | CD1E, CD1C, CD1B, CD1A |
| MF | GO:0030884~exogenous lipid antigen binding | 4 | 2.721 | ＜0.001 | CD1E, CD1C, CD1B, CD1A |
| BP | GO:0048007~antigen processing and presentation, exogenous lipid antigen via MHC class Ib | 4 | 2.721 | ＜0.001 | CD1E, CD1C, CD1B, CD1A |
| CC | GO:0009897~external side of plasma membrane | 14 | 9.524 | ＜0.001 | SEMA7A, CD1E, CD1C, CD1B, LY9, CD1A, IL1RL1, CD209, CD207, S1PR1, ITGAV, FCGR2B, CCR3, CRLF2 |
| BP | GO:0006955~immune response | 14 | 9.524 | ＜0.001 | CCL24, CCL13, SEMA7A, CCL22, CD1E, CD1C, CD1B, PIK3R6, CD1A, IL1RL1, CD209, GPR183, FCGR2B, CCR3 |
| BP | GO:0006954~inflammatory response | 13 | 8.844 | ＜0.001 | CCL24, CCL13, SEMA7A, CCL22, CSF1, ALOX15, IL1RL1, HRH4, FFAR3, FCGR2B, CCL17, CCR3, CCL26 |
| MF | GO:0048020~CCR chemokine receptor binding | 5 | 3.401 | ＜0.001 | CCL24, CCL13, CCL22, CCL17, CCL26 |
| MF | GO:0071723~lipopeptide binding | 4 | 2.721 | ＜0.001 | CD1E, CD1C, CD1B, CD1A |
| BP | GO:0048247~lymphocyte chemotaxis | 5 | 3.401 | ＜0.001 | CCL24, CCL13, CCL22, CCL17, CCL26 |
| BP | GO:0070098~chemokine-mediated signaling pathway | 6 | 4.082 | ＜0.001 | CCL24, CCL13, CCL22, CCL17, CCR3, CCL26 |
| KEGG_PATHWAY | hsa04061:Viral protein interaction with cytokine and cytokine receptor | 7 | 4.762 | ＜0.001 | CCL24, CCL13, CCL22, CSF1, CCL17, CCR3, CCL26 |
| BP | GO:0006935~chemotaxis | 7 | 4.762 | ＜0.001 | CCL24, CCL13, CCL22, S1PR1, CCL17, CCR3, CCL26 |
| BP | GO:0002548~monocyte chemotaxis | 5 | 3.401 | ＜0.001 | CCL24, CCL13, CCL22, CCL17, CCL26 |
| MF | GO:0008009~chemokine activity | 5 | 3.401 | ＜0.001 | CCL24, CCL13, CCL22, CCL17, CCL26 |
| CC | GO:0043231~intracellular membrane-bounded organelle | 17 | 11.565 | ＜0.001 | SMOX, FNBP1, SIAH2, SFMBT2, DAPK2, CTNS, UGT2B28, MIA3, ETV3, CD1C, RUNX3, CD1B, S1PR1, STAP1, CD24, AGFG1, GAPT |
| BP | GO:0070374~positive regulation of ERK1 and ERK2 cascade | 8 | 5.442 | ＜0.001 | CCL24, CCL13, SEMA7A, CCL22, GPR183, ALOX15, CCL17, CCL26 |
| BP | GO:0007165~signal transduction | 20 | 13.605 | ＜0.001 | CCL24, CCL13, CCL22, FNBP1, PDE4D, RHOF, RASAL3, ARHGAP4, IL1RL1, NR4A1, P2RX5, NR4A3, TRAF5, VSTM1, CAMK1, FCGR2B, SIGLEC8, ASB2, RIN2, CCL26 |
| BP | GO:0048245~eosinophil chemotaxis | 4 | 2.721 | ＜0.001 | CCL24, CCL13, CCL22, CCL26 |
| KEGG_PATHWAY | hsa04640:Hematopoietic cell lineage | 6 | 4.082 | 0.001 | CSF1, CD1E, CD24, CD1C, CD1B, CD1A |
| BP | GO:0001938~positive regulation of endothelial cell proliferation | 5 | 3.401 | 0.002 | PPP1R16B, CCL24, NR4A1, CCR3, CCL26 |
| BP | GO:0001916~positive regulation of T cell mediated cytotoxicity | 4 | 2.721 | 0.002 | CD1E, CD1C, CD1B, CD1A |
| BP | GO:0030593~neutrophil chemotaxis | 5 | 3.401 | 0.002 | CCL24, CCL13, CCL22, CCL17, CCL26 |
| BP | GO:0071347~cellular response to interleukin-1 | 5 | 3.401 | 0.002 | CCL24, CCL13, CCL22, CCL17, CCL26 |
| KEGG_PATHWAY | hsa04060:Cytokine-cytokine receptor interaction | 9 | 6.122 | 0.002 | CCL24, IL1RL1, CCL13, CCL22, CSF1, CCL17, CCR3, CCL26, CRLF2 |
| BP | GO:0032754~positive regulation of interleukin-5 production | 3 | 2.041 | 0.003 | IL1RL1, PDE4D, CRLF2 |
| MF | GO:0005515~protein binding | 102 | 69.388 | 0.004 | CSF1, FAM110A, DACH1, CCND2, ZNF329, CPNE2, AGFG1, PPP1R16B, STARD4, ST6GAL1, CISH, FNBP1, DAPK2, SFMBT2, SYTL1, FAM118A, ANXA11, RUNX3, HIC1, FSCN1, SEMA7A, SUCNR1, CALCRL, TTYH2, MAOA, ALOX15, CD1C, RASAL3, CD1B, CD1A, PIK3R6, SOCS2, APH1B, CD300LB, STAP1, CDK17, CPSF7, SIAH2, TMEM120A, HMGA1, PILRB, TVP23A, MEX3C, NR4A1, NR4A3, CD209, SP4, CD207, NOL12, CD24, ITM2A, CLIC4, SIGLEC9, ATP2A3, FHL3, MIA3, SNX10, CHD3, SLC7A11, ARHGAP4, FLVCR1, TNFAIP8L1, FFAR3, ITGAV, TPSAB1, CCR3, DUSP4, PDE4D, ASMTL, ARL4C, GFOD1, IRF4, TFEC, TRAF5, ARHGEF3, CCL13, CEBPE, SATB1, PRR5L, MALT1, IL1RL1, DRAM1, P2RY2, S1PR1, FYN, CTNNAL1, CCL17, CCL24, MARCKSL1, CCL22, BNIP3, SVIP, BCKDK, USH1G, CAMK1, PTPN7, CLC, SIGLEC8, FCGR2B, ASB2, RBM43, CCL26 |
| KEGG_PATHWAY | hsa04062:Chemokine signaling pathway | 7 | 4.762 | 0.004 | CCL24, CCL13, CCL22, CCL17, PIK3R6, CCR3, CCL26 |
| BP | GO:0071346~cellular response to interferon-gamma | 5 | 3.401 | 0.004 | CCL24, CCL13, CCL22, CCL17, CCL26 |
| MF | GO:0046935~1-phosphatidylinositol-3-kinase regulator activity | 3 | 2.041 | 0.005 | SOCS2, CISH, PIK3R6 |
| BP | GO:0043547~positive regulation of GTPase activity | 6 | 4.082 | 0.009 | CCL24, CCL13, CCL22, S1PR1, CCL17, CCL26 |
| CC | GO:0005942~phosphatidylinositol 3-kinase complex | 3 | 2.041 | 0.009 | SOCS2, CISH, PIK3R6 |
| KEGG_PATHWAY | hsa05146:Amoebiasis | 5 | 3.401 | 0.009 | GNA15, CD1E, CD1C, CD1B, CD1A |
| MF | GO:0030246~carbohydrate binding | 6 | 4.082 | 0.013 | SIGLEC9, CD209, LGALS12, CD207, CLC, SIGLEC8 |
| BP | GO:0071356~cellular response to tumor necrosis factor | 5 | 3.401 | 0.013 | CCL24, CCL13, CCL22, CCL17, CCL26 |
| MF | GO:0005537~mannose binding | 3 | 2.041 | 0.014 | CD209, CD207, SLC7A11 |
| CC | GO:0005829~cytosol | 49 | 33.333 | 0.014 | CLIC4, ARHGAP4, FAM110A, DACH1, CCND2, ITGAV, KIF21B, AGFG1, STARD4, CISH, FNBP1, PDE4D, SFMBT2, ASMTL, ANXA11, RHOF, RUNX3, ARL4C, HEATR5A, IRF4, TFEC, TRAF5, ARHGEF3, FSCN1, DXO, RIN2, MAOA, ALOX15, RASAL3, CD1B, MALT1, PIK3R6, SOCS2, IL1RL1, STAP1, FYN, CTNNAL1, PPP1R14A, SMOX, SIAH2, HMGA1, NR4A1, P2RX5, USH1G, SP4, PTPN7, CLC, CAMK1, ASB2 |
| BP | GO:0006897~endocytosis | 6 | 4.082 | 0.015 | HEATR5A, CD209, FNBP1, MIA3, SNX10, RIN2 |
| BP | GO:0006952~defense response | 4 | 2.721 | 0.017 | CEBPE, FCGR2B, TPSAB1, MALT1 |
| BP | GO:0030225~macrophage differentiation | 3 | 2.041 | 0.017 | IL1RL1, CSF1, CEBPE |
| BP | GO:0051482~positive regulation of cytosolic calcium ion concentration involved in phospholipase C-activating G-protein coupled signaling pathway | 3 | 2.041 | 0.019 | GNA15, P2RY10, S1PR1 |
| CC | GO:0031528~microvillus membrane | 3 | 2.041 | 0.019 | SYTL1, ITGAV, SLC7A11 |
| BP | GO:0010730~negative regulation of hydrogen peroxide biosynthetic process | 2 | 1.361 | 0.020 | CTNS, FYN |
| BP | GO:0048021~regulation of melanin biosynthetic process | 2 | 1.361 | 0.020 | CTNS, SLC7A11 |
| MF | GO:0005157~macrophage colony-stimulating factor receptor binding | 2 | 1.361 | 0.020 | CSF1, STAP1 |
| CC | GO:0005887~integral component of plasma membrane | 17 | 11.565 | 0.023 | SEMA7A, P2RY10, SIGLEC9, CALCRL, PILRB, CD1E, CD1C, CD1A, P2RX5, P2RY2, GPR183, SLCO4C1, HRH4, FFAR3, ITGAV, FCGR2B, CCR3 |
| BP | GO:0032743~positive regulation of interleukin-2 production | 3 | 2.041 | 0.024 | IRF4, PDE4D, MALT1 |
| BP | GO:0002313~mature B cell differentiation involved in immune response | 2 | 1.361 | 0.026 | GPR183, FCGR2B |
| CC | GO:0005938~cell cortex | 5 | 3.401 | 0.029 | FAM110A, FNBP1, FSCN1, RHOF, RASAL3 |
| MF | GO:0043565~sequence-specific DNA binding | 7 | 4.762 | 0.031 | MMP12, NR4A1, NR4A3, SATB1, IRF4, SP4, HIC1 |
| BP | GO:0030335~positive regulation of cell migration | 6 | 4.082 | 0.031 | CCL24, SEMA7A, CSF1, S1PR1, ITGAV, CCL26 |
| BP | GO:0032946~positive regulation of mononuclear cell proliferation | 2 | 1.361 | 0.033 | ST6GAL1, CSF1 |
| BP | GO:0071376~cellular response to corticotropin-releasing hormone stimulus | 2 | 1.361 | 0.033 | NR4A1, NR4A3 |
| BP | GO:0030316~osteoclast differentiation | 3 | 2.041 | 0.033 | CSF1, GPR183, SNX10 |
| BP | GO:0035556~intracellular signal transduction | 8 | 5.442 | 0.034 | SOCS2, NR4A3, CISH, CD209, DAPK2, ARHGEF3, FYN, ASB2 |
| MF | GO:0031728~CCR3 chemokine receptor binding | 2 | 1.361 | 0.034 | CCL24, CCL26 |
| BP | GO:0007155~cell adhesion | 9 | 6.122 | 0.034 | SIGLEC9, S1PR1, ITGAV, SLC7A11, CD24, SIGLEC8, CTNNAL1, LY9, CCR3 |
| BP | GO:0022617~extracellular matrix disassembly | 3 | 2.041 | 0.037 | MMP12, TPSAB1, MMP10 |
| BP | GO:0015811~L-cystine transport | 2 | 1.361 | 0.039 | CTNS, SLC7A11 |
| CC | GO:0000785~chromatin | 13 | 8.844 | 0.040 | SATB1, CEBPE, HMGA1, CHD3, ETV3, RUNX3, HIC1, NR4A1, NR4A3, CCND2, IRF4, SP4, TFEC |
| MF | GO:0042802~identical protein binding | 19 | 12.925 | 0.043 | CSF1, FNBP1, CEBPE, DAPK2, BNIP3, FAM118A, RASAL3, MALT1, ARHGAP4, NR4A1, USH1G, SP4, TRAF5, TNFAIP8L1, CD300LB, FYN, CLC, TPSAB1, NOL12 |
| BP | GO:0030838~positive regulation of actin filament polymerization | 3 | 2.041 | 0.045 | CCL24, ALOX15, CCL26 |

**Supplementary Table 6.** Results of GO and KEGG analyses of differentially expressed genes of colon cancer.

| **Category** | **Term** | **Count** | **%** | **P-value** | **Genes** |
| --- | --- | --- | --- | --- | --- |
| MF | GO:0005515~protein binding | 420 | 78.505 | ＜0.001 | SERPINE2, STMN2, TMEM97, CLDN2, SLC4A4, CLDN1, MYLK, SPIB, NSDHL, RNF114, MYC, DPYSL3, MS4A12, IER3, HES6, FNBP1, DNTTIP1, CEL, GTPBP4, SND1, WDR77, SLC5A6, CLDN5, MTHFD2, CLDN8, DDIT4, AGTR1, SPARCL1, CFB, ASF1B, NUP205, RPN2, CDCA3, SHMT2, CDCA5, CDCA7, NOLC1, MRPL17, NCAPG, CDCA8, SLC5A1, THY1, C2, PLAC9, PRDX4, HSPH1, EPB41L3, RRS1, SERPINH1, PPARGC1A, S100A11, PA2G4, COL1A1, SQLE, COL1A2, TCN1, FASN, PSMG1, FERMT2, SRPX, SPIN4, QPRT, JPH1, TMEM47, PCMTD2, TRMT112, LMBR1, PBK, RBPMS2, TIMP1, ENOPH1, DLGAP5, CEP55, RIPK2, TESC, ACTL6A, MAMDC2, BGN, AEN, SERPINB5, GNL3, BACE2, SLC7A5, TUBB2B, MYOT, SLCO4A1, KCNMA1, CKS2, GAS1, ZNF239, NUP62CL, TKT, CRYAB, PAFAH1B3, ZNF593, SF3B3, ODC1, CENPA, SLC7A1, SRPX2, PLAGL2, CKB, CCL19, FLNC, LDLR, JAM2, BCHE, YTHDF1, CENPW, CCL21, CCL20, STAT1, ATAD2, GUCA2B, AGT, GUCA2A, BYSL, CENPF, COL3A1, RFWD3, FOSB, MSRB3, NCAPD2, SCRG1, CDKN3, CSE1L, PDCD5, HJURP, FAM107A, PLOD3, IRS2, YEATS4, BZW2, BEST2, LAPTM4B, CHP2, OIP5, PITX1, PHLDA1, RNF43, SEMA6A, ZBTB33, OLFM4, GTF2F2, CDC25B, ACLY, MELK, NHP2, FSCN1, MAL, SNRPF, S100A9, SNRPB, LY6E, REG3A, DNMT1, TACSTD2, DPT, HMMR, RAD51AP1, STIP1, NDN, BRIX1, IGFBP6, DEFA6, ZBTB16, NEK6, CYBRD1, REEP1, CXCL10, LRCH2, CXCL12, CDK4, NFE2L3, LCN2, CDK1, DHCR7, SLC26A3, HSP90AB1, NXT1, FHL1, RFC4, GPX3, ITGA2, RANGAP1, ADORA2B, POLR1C, MCM3, POLR1D, MCM4, MCM6, MCM2, ST6GALNAC6, CEBPB, UHRF1, PPM1H, ITLN1, TTK, SRM, SELENBP1, DRAM1, CDC45, POLR2D, SH3BP4, E2F3, SLC25A23, E2F5, NOP56, SLC12A2, PPIL1, ANGPT2, SEMA4D, ADRM1, GZMB, MFAP4, SST, PRC1, MGP, PI16, EIF3B, IFITM3, IFITM1, AHCYL2, AQP8, MT1M, CCNF, PDCD2L, CDC20, CHEK1, PDK4, SOX9, EPHB4, SOX4, EPHB3, SMARCC1, SLC11A2, MIF, TM4SF1, SFRP1, LACTB2, MT1F, CCDC86, MT1G, KCTD14, TRIB3, ZG16, KIF20A, MT1E, TMEM63A, MAOA, APCDD1, NEU4, TSPAN7, KCNN4, LYAR, TRPM6, CDT1, TRAP1, TGDS, RCC2, PAICS, CCT6A, TPX2, AKR1B10, CYP2S1, MRPS17, TOP2A, FEN1, SYNM, CITED2, SUV39H1, NAT10, SYNCRIP, UCHL1, ESM1, ENC1, CLDN23, NCS1, CTSG, HTATIP2, CCT3, CCT2, TNFRSF12A, PRMT1, TUBB, PRMT3, PROX1, UCHL3, PDRG1, RPUSD4, VSNL1, KIF2C, NCOA7, MATN2, TAGLN, AHCY, HEATR1, IFI6, GDPD5, NKD1, NKD2, EXOSC7, CST1, EXOSC5, MSX1, LY6G6D, CCT7, CD55, CAP2, GINS2, RANBP1, GDF15, GCG, NPM3, OTOP2, PDZK1IP1, LYVE1, MAD2L2, TRIP13, GRINA, MAD2L1, GOLT1A, TTF2, ETS2, EFTUD2, SCGN, RGS2, CCND1, PLAU, DUOXA2, NUSAP1, SPINK4, PCID2, LMO3, TOMM34, PRPF4, PCP4, COL4A1, DKC1, SCNN1B, TIMELESS, DPEP1, SCG2, RPF2, COX7A1, TMEM123, CNN1, IRAK1, CALD1, MYH11, ECT2, ATP6V1F, TBC1D16, PRPH, STIL, UBE2C, CPSF3, CAV1, RPP40, GGH, HSPA2, BMP7, PYY, BAMBI, UBE2T, KIF4A, TGFBI, GALK1, GPSM2, CXCL9, ZMYND19, HSPB8, CHD7, SLC2A1, CXCL1, CXCL2, LMNB2, CKS1B, POLB, GHR, CA1, CA2, CA4, CA9, MMP7, PTGIS, MMP3, AXIN2, ETV4, MMP9, CCNA2, VCAN, SETBP1, ALDH1A1, CASQ2, COLEC12, LEF1, DDX21, ATP1A2, AURKB, AURKA, PDLIM3, SLIT2, BUB1, CMTM8, RRM2, ARID3A, MEIS1, DES, GNPDA1, SCD, COL7A1, S100P, SSBP1, VIP, ABCE1, GLA, SNTB1, RAN, NUP37 |
| BP | GO:0051301~cell division | 37 | 6.916 | ＜0.001 | GPSM2, CDCA3, CDCA5, CCNF, CDCA7, NCAPG, CDCA8, AURKB, AURKA, CKS1B, CDC20, CCND1, OIP5, BUB1, CDT1, CENPW, UBE2C, NEK6, RCC2, TUBB, CDC25B, MAD2L2, CCNA2, ASPM, TPX2, CENPF, PRC1, KIFC1, CDK4, CKS2, TIMELESS, CDK1, KIF2C, NCAPD2, RAN, NUP37, MAD2L1 |
| CC | GO:0005576~extracellular region | 104 | 19.439 | ＜0.001 | SERPINE2, SCGN, PLAU, LIPG, EPHB4, EPHB3, SPINK4, PCID2, CEL, MIF, OLFM4, ACLY, SFRP1, SFRP2, COL4A1, SPARCL1, MXRA5, S100A9, SCG2, CFB, LY6E, CFD, REG3A, THY1, C2, PLAC9, PRDX4, HSPH1, C7, STC2, PI3, IGFBP6, CLCA4, S100A11, DEFA6, REG4, GGH, PA2G4, BMP7, COL1A1, CXCL10, PYY, AKR1B10, CXCL12, COL1A2, TCN1, COL5A2, LCN2, TGFBI, CXCL9, DHRS11, HSP90AB1, CXCL1, CXCL2, GHR, ESM1, GLIPR2, CTSG, TIMP1, CCT2, MMP7, EDN3, GPX3, TUBB, MAMDC2, MMP3, BGN, MMP9, MMP12, GREM2, MMP11, VCAN, CEACAM7, MATN2, PCOLCE2, FGL2, ITLN1, CLEC3B, ABI3BP, SLIT2, CCL19, LY6G6D, CD55, CTHRC1, CHGA, BCHE, ANGPT2, CCL21, GDF15, CCL20, GUCA2B, GZMB, GCG, GUCA2A, AGT, MFAP5, MFAP4, COL3A1, SST, COL7A1, MGP, S100P, VIP, GLA |
| CC | GO:0005615~extracellular space | 96 | 17.944 | ＜0.001 | CXCL9, SERPINE2, REG1B, REG1A, PLOD3, CXCL1, CXCL2, ACTG2, GHR, GLIPR2, PLAU, ANPEP, DPYSL3, LIPG, CHEK1, CTSG, TIMP1, SEMA6A, MMP7, PTGIS, EDN3, GPX3, MMP3, BGN, CEL, MIF, OLFM4, MMP9, SERPINB5, GNL3, MMP12, GREM2, MMP11, SFRP1, VCAN, SFRP2, COL4A1, MTHFD2, DPEP1, SPARCL1, ZG16, S100A9, SCG2, CFB, COLEC12, CFD, REG3A, FGL2, ITLN1, TACSTD2, DPT, C2, SELENBP1, CST1, CLEC3B, SRPX2, C7, ABI3BP, STC2, SERPINH1, CKB, SLIT2, PI3, IGFBP6, CCL19, S100A11, CTHRC1, CHGA, BCHE, CMTM8, ANGPT2, DEFA6, SEMA4D, CCL21, GDF15, CCL20, GGH, GCG, SULF1, BMP7, AGT, COL1A1, MFAP4, CXCL10, PYY, COL3A1, COL1A2, CILP, TCN1, SST, COL7A1, COL5A2, LCN2, TGFBI, PI16, SCRG1 |
| CC | GO:0070062~extracellular exosome | 104 | 19.439 | ＜0.001 | REG1B, CSE1L, REG1A, PDCD5, PLOD3, SLC4A4, ACTG2, PLAU, ANPEP, EPHB4, CEL, MIF, OLFM4, SND1, ACLY, SFRP1, PPA1, SCNN1B, FSCN1, DPEP1, MXRA5, S100A9, SLC27A2, CFB, CFD, TTYH3, TMEM63A, SHMT2, TACSTD2, SLC5A1, THY1, C2, PRDX4, HSPH1, C7, MYH11, S100A11, ATP6V1F, PRPH, CYBRD1, GGH, PA2G4, HSPA2, PAICS, CCT6A, GGCT, CXCL12, COL1A2, CILP, FASN, LCN2, CDK1, TGFBI, GALK1, HSP90AB1, QPRT, SLC2A1, GPT, CA1, ATIC, GLIPR2, CA2, CA4, CTSG, TIMP1, CCT3, CCT2, MMP7, GPX3, TUBB, CAD, BGN, MMP9, SLC7A5, ALDH1A1, CDH11, TKT, CRYAB, AHCY, HPGD, FGL2, ITLN1, SELENBP1, CLEC3B, SH3BP4, CKB, SLIT2, CCT7, CD55, SLC12A2, GDF15, ATAD2, GUCA2B, PDZK1IP1, LYVE1, AGT, GNPDA1, DES, MGP, S100P, SSBP1, GLA, RAN, EIF3B |
| CC | GO:0005829~cytosol | 202 | 37.757 | ＜0.001 | AHCYL2, SERPINE2, CCNF, STMN2, MYLK, CDC20, RNF114, PPAT, CHEK1, DPYSL3, EPHB4, EPHB3, IER3, FNBP1, MIF, GTPBP4, SND1, WDR77, SFRP1, PPA1, DDIT4, TRIB3, MYL9, ASF1B, NUP205, CDCA3, MAOA, SHMT2, CDCA5, CDCA7, NCAPG, CDCA8, THY1, HSPH1, PRDX4, EPB41L3, KCNN4, PPARGC1A, CDT1, RCC2, PAICS, CCT6A, ANLN, FERMT1, TPX2, AKR1B10, FASN, PSMG1, FERMT2, SPIN4, CITED2, QPRT, GPT, SYNCRIP, TRMT112, UCHL1, ATIC, CTSG, RRP12, ENOPH1, DLGAP5, HTATIP2, CCT3, CCT2, RIPK2, PRMT1, TESC, TUBB, PRMT3, PROX1, UCHL3, SLC7A5, CEACAM7, VSNL1, KIF2C, TKT, CRYAB, PAFAH1B3, AHCY, ADH1C, ADH1B, ODC1, CENPA, EXOSC7, EXOSC5, MTHFD1L, CKB, FLNC, CCT7, RANBP1, STAT1, NPM3, AGT, BYSL, MAD2L2, CENPF, FABP6, MSRB3, CENPN, NCAPD2, CDKN3, MAD2L1, CHPF, GOLT1A, CSE1L, PDCD5, HJURP, IRS2, TTF2, ETS2, ACTG2, EFTUD2, SCGN, RGS2, CCND1, DUOXA2, CHP2, PHLDA1, TMOD1, TPM2, TOMM34, ZBTB33, OLFM4, CDC25B, ACLY, PCP4, FSCN1, SNRPF, S100A9, SLC27A2, SNRPB, TACSTD2, HMMR, STIP1, IRAK1, CALD1, NDN, MYH11, PI3, ECT2, ATP6V1F, TBC1D16, STIL, UBE2C, ZBTB16, NEK6, HSPA2, GGCT, CDK4, KIF4A, TBXAS1, CDK1, GALK1, GPSM2, HSP90AB1, NXT1, HSPB8, FHL1, LMOD1, SLC2A1, RERG, GHR, CA1, CA2, PGM5, GPX2, CKAP2L, CAD, CKAP2, AXIN2, RANGAP1, GPCPD1, CCNA2, TMIGD1, SETBP1, ALDH1A1, POLR1C, POLR1D, HPGD, DDX21, CXXC5, AURKB, SRM, AURKA, SELENBP1, PDLIM3, POLR2D, BUB1, SLC12A2, PPP1R14A, RRM2, ADRM1, GZMB, ARID3A, DES, GNPDA1, PRC1, KLF9, ABCE1, RAN, NUP37, EIF3B |
| KEGG_PATHWAY | hsa04110:Cell cycle | 23 | 4.299 | ＜0.001 | CDT1, CDCA5, TTK, AURKB, CDC25B, CCNA2, MAD2L2, CDC20, CDC45, CCND1, CDK4, MYC, CHEK1, MCM3, CDK1, MCM4, E2F3, TRIP13, MCM6, E2F5, BUB1, MCM2, MAD2L1 |
| MF | GO:0005201~extracellular matrix structural constituent | 19 | 3.551 | ＜0.001 | SRPX, FGL2, BGN, DPT, MFAP5, COL1A1, MFAP4, COL3A1, SRPX2, COL1A2, COL4A1, CILP, ABI3BP, MGP, COL5A2, TGFBI, MXRA5, CTHRC1, MATN2 |
| CC | GO:0005654~nucleoplasm | 154 | 28.785 | ＜0.001 | IFITM2, CSE1L, HJURP, YEATS4, ETS2, EFTUD2, CDC20, CCND1, MYC, CHEK1, CHP2, OIP5, SOX9, PHLDA1, SOX4, SMARCC1, DNTTIP1, GTF3A, ZBTB33, MIF, OLFM4, GTF2F2, GTPBP4, CDC25B, WDR77, ACLY, PRPF4, DKC1, NHP2, TIMELESS, DPEP1, CCDC86, TRIB3, KIF20A, SNRPF, HOXB8, S100A9, ASF1B, SNRPB, DNMT1, CDCA5, CDCA7, NOLC1, CDCA8, RPF2, RAD51AP1, HSPH1, IRAK1, RRS1, NDN, ECT2, LYAR, PPARGC1A, TRAP1, CDT1, STIL, UBE2C, NEK6, CPSF3, RPP40, ANLN, TPX2, CDK4, KIF4A, UBE2T, CDK1, PSMG1, FERMT2, TOP2A, FEN1, NXT1, HSP90AB1, SPIN4, CITED2, SUV39H1, HSPB8, CHD7, NAT10, JPH1, RERG, CKS1B, POLB, TRMT112, SYNCRIP, UCHL1, ENC1, RFC4, PRMT1, TESC, CAD, ACTL6A, AEN, PROX1, RANGAP1, PPRC1, UCHL3, ETV4, CCNA2, RPUSD4, SETBP1, POLR1C, MCM3, POLR1D, MCM4, MCM6, TKT, CRYAB, MCM2, CEBPB, ZNF593, SF3B3, ADH1C, HPGD, ADH1B, UHRF1, HEATR1, LEF1, PPM1H, DDX21, CXXC5, CENPA, AURKB, AURKA, EXOSC7, EXOSC5, CDC45, POLR2D, E2F3, MSX1, E2F5, BUB1, NOP56, CMTM8, GINS2, PPIL1, CENPW, STAT1, ADRM1, ATAD2, ARID3A, NPM3, BYSL, MAD2L2, CENPF, PRC1, RFWD3, KLF9, FOSB, CENPN, S100P, NCAPD2, RAN, NUP37, MAD2L1 |
| BP | GO:0006364~rRNA processing | 18 | 3.364 | ＜0.001 | NOP56, SUV39H1, CHD7, HEATR1, DDX21, NOLC1, DDX10, PA2G4, NPM3, BYSL, EXOSC7, RPUSD4, EXOSC5, DKC1, NHP2, BRIX1, RRP12, LYAR |
| CC | GO:0005694~chromosome | 22 | 4.112 | ＜0.001 | GINS2, CDCA5, DNTTIP1, DDX21, RPF2, ETV4, BYSL, GNL3, RAD51AP1, MAD2L2, RSL1D1, PRC1, KIF4A, NUSAP1, MCM3, CCDC86, MCM4, BRIX1, NCAPD2, TRIP13, MCM6, MCM2 |
| CC | GO:0071162~CMG complex | 6 | 1.121 | ＜0.001 | GINS2, CDC45, MCM3, MCM4, MCM6, MCM2 |
| BP | GO:0006730~one-carbon metabolic process | 9 | 1.682 | ＜0.001 | AHCYL2, CA1, AHCY, MTHFD1L, SHMT2, CA2, MTHFD2, CA4, CA9 |
| CC | GO:0000775~chromosome, centromeric region | 11 | 2.056 | ＜0.001 | TOP2A, CENPF, CENPW, SUV39H1, CDCA5, HJURP, CDCA8, OIP5, KIF2C, CENPA, AURKB |
| BP | GO:0061844~antimicrobial humoral immune response mediated by antimicrobial peptide | 14 | 2.617 | ＜0.001 | REG3A, CXCL9, DEFA6, CCL21, CCL20, REG1B, REG1A, CXCL1, CXCL2, CXCL10, CXCL12, CCL19, VIP, S100A9 |
| BP | GO:0000727~double-strand break repair via break-induced replication | 6 | 1.121 | ＜0.001 | GINS2, CDC45, MCM3, MCM4, MCM6, MCM2 |
| BP | GO:0008284~positive regulation of cell proliferation | 34 | 6.355 | ＜0.001 | REG3A, SHMT2, REG1B, REG1A, ODC1, LEF1, NOLC1, IRS2, TTK, CDC20, ESM1, MYC, CHP2, E2F3, SOX9, TIMP1, SOX4, SMARCC1, EDN3, PRMT1, ACTL6A, MIF, PROX1, CDC25B, WDR77, CLDN5, CXCL10, SFRP1, MEIS1, SFRP2, BAMBI, PRC1, CDK4, VIP |
| BP | GO:0030198~extracellular matrix organization | 18 | 3.364 | ＜0.001 | MMP7, ITGA2, MMP3, MMP9, SERPINB5, MMP12, COL1A1, MMP11, COL3A1, PRDX4, COL1A2, COL4A1, ABI3BP, COL7A1, COL5A2, TGFBI, SOX9, MATN2 |
| CC | GO:0005737~cytoplasm | 191 | 35.701 | ＜0.001 | CSE1L, CCNF, MT1M, PDCD5, STMN2, FAM107A, BZW2, CLDN1, ACTG2, PDCD2L, MYLK, SPIB, SCGN, RGS2, CDH3, CCND1, ANPEP, DPYSL3, CHEK1, NUSAP1, CHP2, PHLDA1, PITX1, SOX4, SMARCC1, PCID2, FNBP1, LMO3, SLC11A2, CEL, MIF, GTPBP4, CDC25B, WDR77, ACLY, PCP4, MELK, PPA1, DKC1, DDIT4, MT1F, FSCN1, MT1G, S100A9, MYL9, MT1E, SNRPB, REG3A, SHMT2, CDCA5, NOLC1, NCAPG, HMMR, NEU4, PRDX4, HSPH1, FGGY, IRAK1, MYH11, ECT2, LYAR, PPARGC1A, S100A11, PRPH, STIL, REG4, NEK6, PA2G4, HSPA2, PAICS, REEP1, CCT6A, COL1A1, FERMT1, TPX2, SMTN, CXCL12, BAMBI, CDK4, KIF4A, CYP2S1, FASN, CDK1, SRPRB, PSMG1, FERMT2, GALK1, TOP2A, GPSM2, NXT1, HSP90AB1, ZMYND19, CITED2, HSPB8, QPRT, FHL1, POLB, PCMTD2, UCHL1, CA1, CA2, ENC1, NCS1, RBPMS2, DACT3, CTSG, DLGAP5, CEP55, HTATIP2, GPX2, RIPK2, PRMT1, TUBB, TESC, CAD, PRMT3, MMP3, CKAP2, AXIN2, PROX1, RANGAP1, UCHL3, GPCPD1, SERPINB5, PDRG1, CCNA2, MMP12, ASPM, TMIGD1, TUBB2B, KIFC1, ALDH1A1, CDH11, MCM3, CASQ2, KIF2C, CRYAB, PAFAH1B3, MCM2, ST6GALNAC6, CEBPB, ZNF593, TAGLN, HPGD, ODC1, LEF1, PPM1H, ADCY3, TTK, ATP1A2, ASCL2, NKD1, NKD2, EXOSC7, EXOSC5, CST1, CLEC3B, DRAM1, CDC45, SRPX2, SH3BP4, FLNC, SLIT2, CCT7, CTHRC1, CAP2, NOP56, RANBP1, PPP1R14A, CMTM8, YTHDF1, GDF15, STAT1, ADRM1, GZMB, NPM3, BYSL, CENPF, GNPDA1, PRC1, FABP6, RFWD3, MSRB3, S100P, NCAPD2, ABCE1, GLA, RAN, SNTB1, CDKN3, MAD2L1 |
| BP | GO:0008283~cell proliferation | 18 | 3.364 | ＜0.001 | EDN3, CITED2, ZBTB16, ITGA2, ODC1, AXIN2, AURKB, BYSL, CKS1B, GNL3, UCHL1, MELK, MYC, LIPG, CKS2, CDK1, TGFBI, BUB1 |
| BP | GO:0007059~chromosome segregation | 12 | 2.243 | ＜0.001 | TOP2A, CDT1, CENPF, CENPW, NEK6, HJURP, TTK, OIP5, CENPN, BUB1, DLGAP5, NUP37 |
| BP | GO:0007094~mitotic spindle assembly checkpoint | 8 | 1.495 | ＜0.001 | MAD2L2, CDC20, CENPF, TTK, TRIP13, BUB1, AURKB, MAD2L1 |
| CC | GO:0000776~kinetochore | 16 | 2.991 | ＜0.001 | CDT1, SMARCC1, CENPW, ACTL6A, HJURP, TTK, RANGAP1, CENPA, AURKB, AURKA, CENPF, KIF2C, CENPN, BUB1, NUP37, MAD2L1 |
| CC | GO:0032991~macromolecular complex | 38 | 7.103 | ＜0.001 | IFITM3, TOP2A, GPSM2, IFITM1, FEN1, IFITM2, HSP90AB1, CITED2, CDCA8, IRS2, SLC7A1, CLDN1, RAD51AP1, POLB, STIP1, TRMT112, HSPH1, IRAK1, MYC, CHEK1, NDN, SOX9, LY6G6D, SMARCC1, PCID2, STAT1, RIPK2, ZBTB16, CAV1, NEK6, TUBB, ACTL6A, OLFM4, PCP4, CRYAB, RAN, SNTB1, ASF1B |
| BP | GO:0006268~DNA unwinding involved in DNA replication | 7 | 1.308 | ＜0.001 | GINS2, CDC45, MCM3, MCM4, SSBP1, MCM6, MCM2 |
| MF | GO:0042802~identical protein binding | 77 | 14.393 | ＜0.001 | GPSM2, HSP90AB1, HSPB8, QPRT, HJURP, SLC2A1, CLDN2, SLC4A4, CLDN1, FGFRL1, LMNB2, GHR, DPYSL3, CLDN23, RBPMS2, DACT3, OIP5, HMGCS2, CEP55, FNBP1, GPX3, RIPK2, PRMT1, TPM2, CAD, MIF, MMP9, GREM2, CLDN5, SFRP1, CLDN8, KCNMA1, TIMELESS, MCM6, CRYAB, PAFAH1B3, REG3A, HPGD, UHRF1, ITLN1, PPM1H, DDX21, TTK, APCDD1, SRM, SRPX2, PRDX4, IRAK1, SH3BP4, SLIT2, FLNC, IGFBP6, LY6G6D, LYAR, LDLR, CAP2, BCHE, STIL, SEMA4D, STAT1, ZBTB16, CAV1, CYBRD1, ARID3A, GCG, PAICS, COL1A1, DES, GNPDA1, COL1A2, SST, PRC1, LCN2, TGFBI, SSBP1, TRIP13, MAD2L1 |
| BP | GO:0007052~mitotic spindle organization | 10 | 1.869 | ＜0.001 | GPSM2, STIL, KIF4A, NEK6, CDCA8, TTK, DLGAP5, AURKB, RAN, AURKA |
| CC | GO:0031526~brush border membrane | 10 | 1.869 | ＜0.001 | SLC5A6, HSP90AB1, AQP8, CA4, SLC11A2, ITLN1, CYBRD1, SLC5A1, TRPM6, SLC26A3 |
| CC | GO:0030496~midbody | 17 | 3.178 | ＜0.001 | RCC2, SLC2A1, CDCA8, NAT10, AURKB, AURKA, GNL3, ASPM, ANLN, CENPF, PRC1, KIF4A, CDK1, KIF20A, ECT2, RAN, CEP55 |
| BP | GO:0031640~killing of cells of other organism | 12 | 2.243 | ＜0.001 | CHGA, CXCL10, CXCL9, CXCL12, DEFA6, C7, CCL21, CCL20, GZMB, CXCL1, CCL19, CXCL2 |
| BP | GO:1904874~positive regulation of telomerase RNA localization to Cajal body | 6 | 1.121 | ＜0.001 | CCT3, CCT6A, CCT2, DKC1, NHP2, CCT7 |
| BP | GO:0043434~response to peptide hormone | 9 | 1.682 | ＜0.001 | COL1A1, REG3A, CXCL12, STAT1, REG1B, STC2, REG1A, TIMP1, BMP7 |
| BP | GO:0000278~mitotic cell cycle | 15 | 2.804 | ＜0.001 | CDT1, CENPW, CDCA5, TUBB, NOLC1, CDCA8, YEATS4, AURKB, CDC25B, AURKA, TPX2, CENPF, TUBB2B, PBK, RAN |
| BP | GO:0000281~mitotic cytokinesis | 10 | 1.869 | ＜0.001 | ANLN, KIF4A, NUSAP1, CKAP2, CDCA8, KIF20A, ECT2, CENPA, AURKB, CEP55 |
| CC | GO:0005634~nucleus | 196 | 36.636 | ＜0.001 | CCNF, MT1M, SPIB, RNF114, MYC, CHEK1, SOX9, SOX4, IER3, HES6, SMARCC1, SLC11A2, DNTTIP1, DDX10, GTPBP4, SND1, WDR77, MT1F, CCDC86, MT1G, TRIB3, KIF20A, HOXB8, MT1E, ASF1B, SHMT2, CDCA5, CDCA7, NCAPG, HSPH1, PRDX4, PPARGC1A, LYAR, NKX2-3, S100A11, CDT1, RCC2, PA2G4, TPX2, PSMG1, FERMT2, TOP2A, FEN1, CITED2, SUV39H1, NAT10, SYNCRIP, ENC1, PBK, CTSG, RRP12, ENOPH1, DLGAP5, HTATIP2, PRMT1, TESC, TUBB, ACTL6A, PRMT3, AEN, PROX1, PPRC1, GNL3, MMP12, TUBB2B, KIFC1, ZNF239, KIF2C, NCOA7, CRYAB, SF3B3, CENPA, ASCL2, EXOSC7, EXOSC5, TCEAL2, PLAGL2, CKB, MSX1, RANBP1, GINS2, STAT1, GDF15, ATAD2, MAD2L2, CENPF, FABP6, RFWD3, FOSB, NCAPD2, TRIP13, CDKN3, MAD2L1, CSE1L, PDCD5, HJURP, FAM107A, YEATS4, TTF2, ETS2, FOXQ1, EFTUD2, SCGN, RGS2, CCND1, NUSAP1, CHP2, OIP5, PITX1, PHLDA1, PCID2, LMO3, TOMM34, GTF3A, ZBTB33, PUS7, GTF2F2, CDC25B, PRPF4, PCP4, DKC1, TIMELESS, SNRPF, S100A9, SNRPB, DNMT1, TACSTD2, RAD51AP1, STIP1, IRAK1, NDN, ECT2, UBE2C, ZBTB16, NEK6, CPSF3, RPP40, GGH, HSPA2, CXCL12, CDK4, UBE2T, NFE2L3, CDK1, HSP90AB1, NXT1, HSPB8, FHL1, CHD7, RERG, GHR, POLB, PTGIS, RFC4, PEG3, ITGA2, MMP3, CAD, AXIN2, RANGAP1, ETV4, CCNA2, ASPM, POLR1C, MCM3, MCM4, MCM6, MCM2, CEBPB, UHRF1, LEF1, PPM1H, TTK, CXXC5, AURKB, AURKA, CDC45, POLR2D, SH3BP4, E2F3, E2F5, BUB1, PPIL1, RRM2, ANGPT2, ADRM1, GZMB, ARID3A, MEIS1, DES, PRC1, KLF9, S100P, SSBP1, RAN, NUP37 |
| BP | GO:0010718~positive regulation of epithelial to mesenchymal transition | 9 | 1.682 | ＜0.001 | MAD2L2, COL1A1, GLIPR2, BAMBI, LEF1, AXIN2, BMP7, FERMT2, AGT |
| BP | GO:0034080~CENP-A containing nucleosome assembly | 5 | 0.935 | ＜0.001 | CENPW, HJURP, OIP5, CENPN, CENPA |
| BP | GO:1904851~positive regulation of establishment of protein localization to telomere | 5 | 0.935 | ＜0.001 | CCT3, CCT6A, CCT2, DKC1, CCT7 |
| BP | GO:1904871~positive regulation of protein localization to Cajal body | 5 | 0.935 | ＜0.001 | CCT3, CCT6A, CCT2, DKC1, CCT7 |
| MF | GO:0008307~structural constituent of muscle | 8 | 1.495 | ＜0.001 | SYNM, SMTN, MYOT, TPM2, NEXN, MYH11, JPH1, MYL9 |
| BP | GO:0071230~cellular response to amino acid stimulus | 9 | 1.682 | ＜0.001 | COL1A1, COL3A1, DNMT1, CEBPB, COL1A2, COL4A1, COL5A2, SH3BP4, HMGCS2 |
| BP | GO:0009410~response to xenobiotic stimulus | 19 | 3.551 | ＜0.001 | BCHE, HSP90AB1, RPN2, STAT1, ITGA2, COL1A1, CENPF, SFRP1, CDH3, SFRP2, CCND1, CDK4, SST, MYC, SCNN1B, CDK1, FOSB, CA9, HMGCS2 |
| BP | GO:0030199~collagen fibril organization | 10 | 1.869 | ＜0.001 | COL1A1, MMP11, COL3A1, SFRP2, COL1A2, COL4A1, COL5A2, SERPINH1, PLOD3, DPT |
| BP | GO:0042127~regulation of cell proliferation | 15 | 2.804 | ＜0.001 | CXCL9, STAT1, CDCA7, AGT, GNL3, CXCL10, SQLE, TMIGD1, PLAU, CDK4, CHEK1, AGTR1, SOX9, NKX2-3, S100A11 |
| KEGG_PATHWAY | hsa04978:Mineral absorption | 10 | 1.869 | ＜0.001 | MT1M, SLC11A2, MT1F, CYBRD1, MT1G, ATP1A2, SLC5A1, TRPM6, SLC26A3, MT1E |
| BP | GO:0030879~mammary gland development | 6 | 1.121 | ＜0.001 | FASN, ITGA2, CAV1, LEF1, IRS2, SOX9 |
| BP | GO:0001501~skeletal system development | 13 | 2.430 | ＜0.001 | CHD7, BMP7, MMP9, ETS2, FGFRL1, COL1A1, VCAN, COL1A2, COL5A2, CDH11, SOX9, PITX1, SOX4 |
| CC | GO:0016323~basolateral plasma membrane | 18 | 3.364 | ＜0.001 | SLC12A2, HSP90AB1, HPGD, AQP8, CAV1, SLC2A1, ABCA8, SLC7A1, CLDN1, SLC4A4, NKD2, AURKA, SLC5A6, SLC7A5, CLDN8, SLCO1B3, CA9, LDLR |
| CC | GO:0072686~mitotic spindle | 13 | 2.430 | ＜0.001 | CKAP2L, TUBB, CKAP2, RANGAP1, AURKA, TPX2, TUBB2B, KIFC1, NUSAP1, CDK1, ECT2, DLGAP5, MAD2L1 |
| BP | GO:0001503~ossification | 10 | 1.869 | ＜0.001 | COL1A1, CLEC3B, IFITM1, MGP, CDH11, COL5A2, SOX9, MMP9, BMP7, GPM6B |
| BP | GO:0030593~neutrophil chemotaxis | 10 | 1.869 | ＜0.001 | CXCL10, CXCL9, EDN3, CCL21, CCL20, DPEP1, CXCL1, CCL19, S100A9, CXCL2 |
| MF | GO:0042803~protein homodimerization activity | 38 | 7.103 | ＜0.001 | TOP2A, CEBPB, HSP90AB1, SHMT2, HSPB8, ODC1, SRM, GHR, ATIC, IRAK1, MTHFD1L, GLIPR2, STC2, GRPEL2, RBPMS2, SLIT2, ECT2, S100A11, RRM2, DEFA6, GDF15, STAT1, RIPK2, TPM2, TESC, ZBTB16, DNTTIP1, GGCT, TMIGD1, CENPF, CASQ2, S100P, SSBP1, TKT, CRYAB, GLA, PAFAH1B3, MAD2L1 |
| BP | GO:0030174~regulation of DNA-dependent DNA replication initiation | 5 | 0.935 | ＜0.001 | CDT1, MCM3, MCM4, MCM6, MCM2 |
| CC | GO:0042383~sarcolemma | 11 | 2.056 | ＜0.001 | SYNM, DES, MYOT, CAV1, SLC2A1, SSPN, BGN, PGM5, FLNC, SNTB1, RYR3 |
| MF | GO:0008009~chemokine activity | 8 | 1.495 | ＜0.001 | CXCL10, CXCL9, CXCL12, CCL21, CCL20, CXCL1, CCL19, CXCL2 |
| CC | GO:0005819~spindle | 13 | 2.430 | ＜0.001 | TTK, HMMR, AURKB, AURKA, MAD2L2, CDC20, ASPM, TPX2, CENPF, PRC1, NUSAP1, KIF2C, KIF20A |
| MF | GO:0008201~heparin binding | 15 | 2.804 | ＜0.001 | PCOLCE2, SERPINE2, REG4, PDCD5, CEL, BMP7, FGFRL1, GREM2, CXCL10, CLEC3B, SFRP1, ABI3BP, LIPG, CTSG, SLIT2 |
| BP | GO:0051382~kinetochore assembly | 5 | 0.935 | ＜0.001 | CENPF, CENPW, CENPN, CENPA, DLGAP5 |
| CC | GO:0030018~Z disc | 12 | 2.243 | ＜0.001 | PDLIM3, DES, MYOT, SLC2A1, CASQ2, NEXN, PGM5, FLNC, JPH1, CRYAB, MYL9, RYR3 |
| CC | GO:0016324~apical plasma membrane | 23 | 4.299 | ＜0.001 | SLC12A2, HSP90AB1, AQP8, CAV1, SLC11A2, SLC2A1, CYBRD1, SLC5A1, THY1, SLC7A1, CLDN1, SLC5A6, ASPM, SLC7A5, CEACAM7, SCNN1B, KCNMA1, CA4, DPEP1, MAL, TRPM6, SLC26A3, CLCA4 |
| BP | GO:0006915~apoptotic process | 32 | 5.981 | ＜0.001 | PDCD5, CDCA7, IFI6, PDCD2L, AURKA, DRAM1, CHEK1, EPB41L3, PHLDA1, BUB1, IER3, HTATIP2, SEMA6A, TNFRSF12A, PEG3, RIPK2, ZBTB16, NEK6, CKAP2, GZMB, SULF1, MMP9, TPX2, SFRP2, MELK, DDIT4, CDK1, LCN2, DPEP1, MAL, S100A9, MCM2 |
| BP | GO:0001649~osteoblast differentiation | 12 | 2.243 | ＜0.001 | COL1A1, SYNCRIP, RSL1D1, SFRP1, VCAN, FASN, LEF1, DDX21, AXIN2, SND1, GTPBP4, CTHRC1 |
| BP | GO:0008285~negative regulation of cell proliferation | 26 | 4.860 | ＜0.001 | IFITM1, SERPINE2, PDCD5, DPT, CXCL1, FGFRL1, RERG, ABI3BP, NDN, SH3BP4, MSX1, IGFBP6, SOX4, S100A11, BCHE, TESC, ZBTB16, AXIN2, PROX1, BMP7, GTPBP4, SFRP1, SFRP2, SST, FABP6, CDKN3 |
| CC | GO:0000779~condensed chromosome, centromeric region | 5 | 0.935 | ＜0.001 | CEBPB, NCAPG, NCAPD2, CENPA, AURKB |
| MF | GO:0045236~CXCR chemokine receptor binding | 5 | 0.935 | ＜0.001 | CXCL10, CXCL9, CXCL12, CXCL1, CXCL2 |
| CC | GO:0005581~collagen trimer | 10 | 1.869 | ＜0.001 | COLEC12, COL1A1, COL3A1, COL1A2, COL4A1, COL7A1, COL5A2, SERPINH1, TIMP1, CTHRC1 |
| MF | GO:0016887~ATPase activity | 25 | 4.673 | ＜0.001 | HSP90AB1, CHD7, DDX21, ATP1A2, HSPH1, CCT7, CCT3, CCT2, TRAP1, RFC4, ATAD2, DDX10, HSPA2, ABCA8, GTF2F2, CCT6A, KIFC1, MCM3, MCM4, KIF2C, KIF20A, MCM6, TRIP13, ABCE1, MCM2 |
| CC | GO:0000307~cyclin-dependent protein kinase holoenzyme complex | 7 | 1.308 | ＜0.001 | CCNA2, CCND1, CDK4, CCNF, CDK1, CKS2, CKS1B |
| BP | GO:0007049~cell cycle | 22 | 4.112 | ＜0.001 | CDT1, CDCA3, UHRF1, CDCA5, RCC2, SUV39H1, FAM107A, HJURP, PDCD2L, CDC25B, AURKA, CKS1B, RGS2, CDC45, MCM3, MCM4, GAS1, OIP5, MCM6, CDKN3, MCM2, NUP37 |
| BP | GO:0010971~positive regulation of G2/M transition of mitotic cell cycle | 6 | 1.121 | ＜0.001 | CCND1, CDK4, RCC2, CDK1, HSPA2, CDC25B |
| CC | GO:0042555~MCM complex | 4 | 0.748 | ＜0.001 | MCM3, MCM4, MCM6, MCM2 |
| BP | GO:0032092~positive regulation of protein binding | 9 | 1.682 | 0.001 | CLDN5, HSP90AB1, BAMBI, RIPK2, CAV1, TRIB3, GCG, RAN, CTHRC1 |
| CC | GO:0031012~extracellular matrix | 17 | 3.178 | 0.001 | COLEC12, MMP7, MMP3, BGN, MMP12, COL1A1, MMP11, COL3A1, VCAN, COL1A2, COL4A1, MGP, COL5A2, TIMP1, TGFBI, PI3, MATN2 |
| BP | GO:0007051~spindle organization | 5 | 0.935 | 0.001 | ASPM, RANBP1, TTK, AURKB, AURKA |
| BP | GO:0001666~response to hypoxia | 13 | 2.430 | 0.001 | ANGPT2, CITED2, ITGA2, CAV1, SLC11A2, SLC2A1, ASCL2, CXCL12, PLAU, KCNMA1, DDIT4, CA9, CRYAB |
| CC | GO:0015630~microtubule cytoskeleton | 14 | 2.617 | 0.001 | CKAP2L, SHMT2, TUBB, CKAP2, CDCA8, HMMR, GTF2F2, AURKB, AURKA, TPX2, TUBB2B, PRC1, KIF2C, CRYAB |
| KEGG_PATHWAY | hsa03030:DNA replication | 7 | 1.308 | 0.001 | FEN1, RFC4, MCM3, MCM4, SSBP1, MCM6, MCM2 |
| BP | GO:0006260~DNA replication | 11 | 2.056 | 0.002 | POLB, GINS2, FEN1, RFC4, RRM2, CHEK1, CDK1, MCM4, SSBP1, MCM6, MCM2 |
| BP | GO:0097421~liver regeneration | 6 | 1.121 | 0.002 | SLC7A5, CEBPB, CCND1, MYC, CLDN1, AURKA |
| KEGG_PATHWAY | hsa04657:IL-17 signaling pathway | 11 | 2.056 | 0.002 | CXCL10, CEBPB, HSP90AB1, CCL20, MMP3, LCN2, FOSB, CXCL1, S100A9, CXCL2, MMP9 |
| BP | GO:0032212~positive regulation of telomere maintenance via telomerase | 6 | 1.121 | 0.002 | CCT3, CCT6A, CCT2, DKC1, CCT7, AURKB |
| BP | GO:0048144~fibroblast proliferation | 6 | 1.121 | 0.002 | COL3A1, CAV1, CDK1, CKS2, AGT, CKS1B |
| MF | GO:0008327~methyl-CpG binding | 6 | 1.121 | 0.002 | DNMT1, UHRF1, PRMT1, ZBTB33, CXXC5, WDR77 |
| BP | GO:0071560~cellular response to transforming growth factor beta stimulus | 8 | 1.495 | 0.002 | COL1A1, CLEC3B, SFRP1, CILP, CAV1, PDCD5, SOX9, CLDN1 |
| BP | GO:0030308~negative regulation of cell growth | 11 | 2.056 | 0.002 | SFRP1, SFRP2, SERPINE2, FHL1, DACT3, SH3BP4, MSX1, SLIT2, CRYAB, AGT, RERG |
| BP | GO:0016338~calcium-independent cell-cell adhesion via plasma membrane cell-adhesion molecules | 5 | 0.935 | 0.002 | CLDN5, CLDN8, CLDN23, CLDN2, CLDN1 |
| BP | GO:0002009~morphogenesis of an epithelium | 5 | 0.935 | 0.002 | CA2, TIMELESS, CA9, SOX9, SERPINB5 |
| BP | GO:0045471~response to ethanol | 10 | 1.869 | 0.002 | POLB, CLDN5, RGS2, HPGD, CCND1, MYC, TBXAS1, CDK1, HMGCS2, CLDN1 |
| CC | GO:0005832~chaperonin-containing T-complex | 4 | 0.748 | 0.002 | CCT3, CCT6A, CCT2, CCT7 |
| MF | GO:0016836~hydro-lyase activity | 5 | 0.935 | 0.002 | CA1, CA2, CYP2S1, CA4, CA9 |
| BP | GO:0006936~muscle contraction | 9 | 1.682 | 0.002 | TMOD1, DES, CALD1, MYOT, TPM2, SSPN, LMOD1, CRYAB, SNTB1 |
| MF | GO:0005518~collagen binding | 8 | 1.495 | 0.002 | MMP12, PCOLCE2, ABI3BP, ITGA2, SERPINH1, SPARCL1, TGFBI, MMP9 |
| BP | GO:0071711~basement membrane organization | 5 | 0.935 | 0.003 | MMP11, FERMT1, COL3A1, COL4A1, CAV1 |
| BP | GO:0007155~cell adhesion | 28 | 5.234 | 0.003 | SRPX, DPT, NEXN, THY1, CLDN2, CLDN1, CDH3, CLDN23, PGM5, EPHB4, TNFRSF12A, SEMA4D, ITGA2, OLFM4, LYVE1, COL1A1, MFAP4, CLDN5, FERMT1, VCAN, CXCL12, MYOT, COL7A1, CLDN8, CDH11, SSPN, TGFBI, FERMT2 |
| BP | GO:0070098~chemokine-mediated signaling pathway | 8 | 1.495 | 0.003 | CXCL10, CXCL9, CXCL12, CCL21, CCL20, CXCL1, CCL19, CXCL2 |
| MF | GO:0042393~histone binding | 14 | 2.617 | 0.003 | SMARCC1, UHRF1, STAT1, ATAD2, LEF1, CHD7, HJURP, YEATS4, NPM3, CKS1B, CKS2, NCAPD2, MCM2, ASF1B |
| BP | GO:0051256~mitotic spindle midzone assembly | 4 | 0.748 | 0.003 | PRC1, KIF4A, CDCA8, AURKB |
| BP | GO:0035456~response to interferon-beta | 4 | 0.748 | 0.003 | IFITM3, IFITM1, IFITM2, STAT1 |
| KEGG_PATHWAY | hsa04270:Vascular smooth muscle contraction | 13 | 2.430 | 0.003 | PPP1R14A, EDN3, ADCY3, ACTG2, AGT, MYLK, CALD1, ADORA2B, KCNMB1, KCNMA1, AGTR1, MYH11, MYL9 |
| BP | GO:0006913~nucleocytoplasmic transport | 7 | 1.308 | 0.003 | RANBP1, NUP205, NXT1, RANGAP1, NUP62CL, RAN, NUP37 |
| BP | GO:0030178~negative regulation of Wnt signaling pathway | 7 | 1.308 | 0.003 | RNF43, SFRP1, SFRP2, DACT3, APCDD1, NKD1, NKD2 |
| MF | GO:0048407~platelet-derived growth factor binding | 4 | 0.748 | 0.003 | COL1A1, COL3A1, COL1A2, COL4A1 |
| MF | GO:0003682~chromatin binding | 25 | 4.673 | 0.003 | TOP2A, CEBPB, DNMT1, CITED2, SHMT2, CDCA5, SUV39H1, CHD7, LEF1, CENPA, CDC45, SOX9, CDT1, SMARCC1, ACTL6A, ATAD2, ARID3A, NPM3, CENPF, MEIS1, UBE2T, CKS2, CDK1, SSBP1, RAN |
| CC | GO:0045171~intercellular bridge | 9 | 1.682 | 0.003 | TPX2, TUBB2B, PRC1, TUBB, CDCA8, KIF20A, FLNC, OLFM4, CEP55 |
| MF | GO:0045296~cadherin binding | 19 | 3.551 | 0.003 | NOP56, RANBP1, HSP90AB1, STAT1, OLFM4, BZW2, RANGAP1, PAICS, SND1, ANLN, RSL1D1, CDH3, ATIC, CALD1, FASN, CDH11, FSCN1, S100P, RAN |
| MF | GO:0051082~unfolded protein binding | 11 | 2.056 | 0.003 | CCT3, CCT6A, CCT2, TRAP1, HSP90AB1, GRPEL2, SERPINH1, HSPA2, CCT7, CRYAB, PDRG1 |
| BP | GO:0090267~positive regulation of mitotic cell cycle spindle assembly checkpoint | 4 | 0.748 | 0.003 | PCID2, CDCA8, AURKB, MAD2L1 |
| BP | GO:0044772~mitotic cell cycle phase transition | 5 | 0.935 | 0.003 | CCNA2, CCND1, CCNF, CKS2, CKS1B |
| CC | GO:0034709~methylosome | 4 | 0.748 | 0.004 | PRMT1, SNRPF, SNRPB, WDR77 |
| KEGG_PATHWAY | hsa04976:Bile secretion | 10 | 1.869 | 0.004 | CA2, AQP8, SLCO1B3, SLC2A1, UGT2B17, ADCY3, ATP1A2, SLC5A1, LDLR, SLC4A4 |
| BP | GO:0071353~cellular response to interleukin-4 | 5 | 0.935 | 0.004 | HSP90AB1, CDK4, FASN, LEF1, MCM2 |
| BP | GO:0044278~cell wall disruption in other organism | 3 | 0.561 | 0.004 | REG3A, REG1B, REG1A |
| BP | GO:0030336~negative regulation of cell migration | 13 | 2.430 | 0.004 | IFITM1, CITED2, MIF, THY1, SULF1, GTPBP4, CLDN5, SFRP1, SFRP2, DPYSL3, CDH11, DPEP1, SLIT2 |
| BP | GO:0045597~positive regulation of cell differentiation | 7 | 1.308 | 0.004 | GHR, SMARCC1, HSP90AB1, EDN3, ACTL6A, LEF1, PA2G4 |
| BP | GO:0071248~cellular response to metal ion | 4 | 0.748 | 0.004 | MT1M, MT1F, MT1G, MT1E |
| CC | GO:0005874~microtubule | 18 | 3.364 | 0.004 | CCT3, CCT2, RCC2, TUBB, NEK6, CKAP2, AURKA, CCT6A, POLB, TPX2, TUBB2B, HSPH1, KIFC1, KIF4A, NUSAP1, KIF2C, KIF20A, CCT7 |
| MF | GO:0070492~oligosaccharide binding | 4 | 0.748 | 0.005 | REG3A, REG1B, REG1A, ITLN1 |
| CC | GO:0016363~nuclear matrix | 10 | 1.869 | 0.005 | CENPF, SMARCC1, CEBPB, CENPW, UHRF1, KIF4A, ACTL6A, CAD, ENC1, YEATS4 |
| BP | GO:0010564~regulation of cell cycle process | 4 | 0.748 | 0.006 | SFRP1, MYC, SOX9, SND1 |
| BP | GO:0030239~myofibril assembly | 4 | 0.748 | 0.006 | TMOD1, LMOD1, PGM5, MYL9 |
| CC | GO:0005876~spindle microtubule | 6 | 1.121 | 0.006 | POLB, PRC1, KIF4A, CDK1, AURKB, AURKA |
| BP | GO:0090090~negative regulation of canonical Wnt signaling pathway | 12 | 2.243 | 0.006 | MAD2L2, FERMT1, SFRP1, SFRP2, CAV1, DACT3, IGFBP6, SOX9, AXIN2, NKD1, NKD2, CTHRC1 |
| CC | GO:0009986~cell surface | 29 | 5.421 | 0.006 | SRPX, HSP90AB1, HMMR, THY1, SLC4A4, GHR, SRPX2, IRAK1, PLAU, LIPG, CA4, CTSG, SLIT2, LDLR, CD55, JAM2, SCARA5, ITGA2, SLC11A2, BGN, CEL, HSPA2, MIF, SULF1, TMIGD1, SFRP1, CEACAM7, CRYAB, FERMT2 |
| CC | GO:0005788~endoplasmic reticulum lumen | 17 | 3.178 | 0.006 | BCHE, PCID2, PLOD3, GCG, COL1A1, COL3A1, VCAN, COL1A2, COL4A1, STC2, COL7A1, COL5A2, SERPINH1, SPARCL1, TIMP1, SCG2, SLC27A2 |
| BP | GO:0006270~DNA replication initiation | 5 | 0.935 | 0.006 | CDC45, MCM3, MCM4, MCM6, MCM2 |
| CC | GO:0031429~box H/ACA snoRNP complex | 3 | 0.561 | 0.006 | DKC1, NHP2, NOLC1 |
| CC | GO:0016020~membrane | 117 | 21.869 | 0.007 | IFITM1, GOLT1A, CSE1L, STMN2, BZW2, CLDN1, SLC4A4, PDCD2L, EFTUD2, CDH3, CCND1, VSIG2, DUOXA2, IER3, TMOD1, SEMA6A, PAQR4, SLC11A2, TOMM34, UGT2B17, GTPBP4, SND1, SLC5A6, ACLY, CLDN5, MELK, COL4A1, AGTR1, NUP205, RPN2, TACSTD2, NCAPG, HMMR, TMEM123, NEU4, ATP6V1F, PRPH, TRAP1, CAV1, RCC2, CYBRD1, PA2G4, HSPA2, PAICS, REEP1, SQLE, KIF4A, FASN, CDK1, SRPRB, DHCR7, SLC26A3, GALK1, LRP11, SRPX, FEN1, HSP90AB1, ZMYND19, SLC2A1, LMOD1, NAT10, RERG, RYR3, GHR, SYNCRIP, LMBR1, ATIC, CA4, CA9, CTSG, CEP55, HTATIP2, CAD, MAMDC2, GNL3, SLC7A5, BACE2, VCAN, VSNL1, KIFC1, KCNMA1, CDH11, MCM3, GAS1, MCM4, KIF2C, PAFAH1B3, ST6GALNAC6, COLEC12, HEATR1, ADCY3, DDX21, TTK, ATP1A2, SLC7A1, SELENBP1, MTHFD1L, SLIT2, LDLR, BUB1, NOP56, SLC12A2, CMTM8, GZMB, PDZK1IP1, LYVE1, BYSL, RSL1D1, DPY19L1, TMEM158, FABP6, SCD, NCAPD2, ABCE1, RAN, SNTB1, GRINA |
| BP | GO:0051216~cartilage development | 7 | 1.308 | 0.007 | ZBTB16, MGP, TIMP1, SOX9, SULF1, PITX1, BMP7 |
| BP | GO:0071504~cellular response to heparin | 3 | 0.561 | 0.007 | SFRP1, SOX9, SLIT2 |
| BP | GO:0000320~re-entry into mitotic cell cycle | 3 | 0.561 | 0.007 | CCND1, MYC, CCNF |
| BP | GO:1902975~mitotic DNA replication initiation | 3 | 0.561 | 0.007 | MCM3, MCM4, MCM2 |
| BP | GO:0097435~supramolecular fiber organization | 4 | 0.748 | 0.007 | MFAP5, MFAP4, COL3A1, HSP90AB1 |
| MF | GO:0005524~ATP binding | 60 | 11.215 | 0.007 | TOP2A, HSP90AB1, CHD7, NAT10, TTF2, ACTG2, MYLK, CHEK1, PDK4, PBK, EPHB4, EPHB3, CCT3, CCT2, RFC4, RIPK2, CAD, DDX10, GTF2F2, ACLY, MELK, KIFC1, MCM3, MCM4, TRIB3, KIF2C, KIF20A, MCM6, SLC27A2, MCM2, NOLC1, DDX21, ADCY3, ATP1A2, TTK, AURKB, AURKA, HSPH1, MTHFD1L, IRAK1, MYH11, CKB, CCT7, TRPM6, BUB1, TRAP1, UBE2C, NEK6, ATAD2, HSPA2, ABCA8, PAICS, CCT6A, CDK4, UBE2T, KIF4A, CDK1, TRIP13, ABCE1, GALK1 |
| MF | GO:0003779~actin binding | 19 | 3.551 | 0.007 | TMOD1, TAGLN, TPM2, FAM107A, LMOD1, MYLK, ANLN, CNN1, SMTN, PDLIM3, CALD1, MYOT, EPB41L3, KCNMA1, ENC1, FSCN1, FERMT2, SNTB1, CAP2 |
| BP | GO:0048041~focal adhesion assembly | 5 | 0.935 | 0.007 | RCC2, ITGA2, CDH11, THY1, FERMT2 |
| BP | GO:0071347~cellular response to interleukin-1 | 8 | 1.495 | 0.007 | SFRP1, CEBPB, PTGIS, CCL21, CCL20, MYC, SOX9, CCL19 |
| MF | GO:0034513~box H/ACA snoRNA binding | 3 | 0.561 | 0.007 | DKC1, NHP2, NOLC1 |
| BP | GO:0043066~negative regulation of apoptotic process | 25 | 4.673 | 0.007 | HSP90AB1, CITED2, LEF1, IFI6, AURKA, MYC, SOX9, MSX1, TIMP1, IER3, HTATIP2, STIL, PCID2, SEMA4D, GCG, PA2G4, MIF, MMP9, TMIGD1, SFRP1, CDK1, DPEP1, VIP, CRYAB, MAD2L1 |
| BP | GO:0022617~extracellular matrix disassembly | 6 | 1.121 | 0.007 | MMP12, MMP11, MMP7, MMP3, CTSG, MMP9 |
| MF | GO:0030020~extracellular matrix structural constituent conferring tensile strength | 6 | 1.121 | 0.008 | COL1A1, COL3A1, COL1A2, COL4A1, COL7A1, COL5A2 |
| CC | GO:0032133~chromosome passenger complex | 3 | 0.561 | 0.009 | CDCA8, AURKB, AURKA |
| BP | GO:0010629~negative regulation of gene expression | 17 | 3.178 | 0.009 | DNMT1, CITED2, MIF, AGT, AURKA, SLC7A5, CLDN5, FERMT1, SFRP1, SFRP2, CILP, MYC, STC2, CDK1, SOX9, CRYAB, LDLR |
| BP | GO:0010273~detoxification of copper ion | 4 | 0.748 | 0.010 | MT1M, MT1F, MT1G, MT1E |
| BP | GO:0006189~'de novo' IMP biosynthetic process | 3 | 0.561 | 0.010 | ATIC, PPAT, PAICS |
| BP | GO:0009113~purine nucleobase biosynthetic process | 3 | 0.561 | 0.010 | SHMT2, PPAT, PAICS |
| BP | GO:0060414~aorta smooth muscle tissue morphogenesis | 3 | 0.561 | 0.010 | COL3A1, PROX1, MYLK |
| MF | GO:0016538~cyclin-dependent protein serine/threonine kinase regulator activity | 5 | 0.935 | 0.010 | CCNA2, CCND1, CDK4, CCNF, CKS1B |
| CC | GO:0001725~stress fiber | 7 | 1.308 | 0.010 | PDLIM3, FSCN1, FAM107A, PGM5, MYL9, FERMT2, MYLK |
| MF | GO:0004089~carbonate dehydratase activity | 4 | 0.748 | 0.011 | CA1, CA2, CA4, CA9 |
| BP | GO:0032355~response to estradiol | 8 | 1.495 | 0.011 | GHR, COL1A1, HPGD, CCND1, MYC, CRYAB, BMP7, AGT |
| CC | GO:0045177~apical part of cell | 7 | 1.308 | 0.011 | CA2, AQP8, SLC11A2, DUOXA2, DPEP1, LDLR, BYSL |
| CC | GO:0001673~male germ cell nucleus | 6 | 1.121 | 0.011 | TOP2A, SMARCC1, ZBTB16, TRIP13, HSPA2, RAN |
| BP | GO:0070830~bicellular tight junction assembly | 6 | 1.121 | 0.011 | CLDN5, CLDN8, CLDN23, CLDN2, ECT2, CLDN1 |
| BP | GO:0050678~regulation of epithelial cell proliferation | 4 | 0.748 | 0.011 | UHRF1, TACSTD2, NKX2-3, SERPINB5 |
| BP | GO:0007076~mitotic chromosome condensation | 4 | 0.748 | 0.011 | CDCA5, NUSAP1, NCAPG, NCAPD2 |
| BP | GO:0006366~transcription from RNA polymerase II promoter | 14 | 2.617 | 0.012 | CEBPB, LEF1, CHD7, DDX21, PROX1, GTF2F2, MYC, POLR2D, NFE2L3, FOSB, SOX9, TRIP13, MSX1, NKX2-3 |
| KEGG_PATHWAY | hsa05166:Human T-cell leukemia virus 1 infection | 16 | 2.991 | 0.012 | RANBP1, MMP7, SLC2A1, ADCY3, ETS2, POLB, CCNA2, CDC20, CCND1, CDK4, MYC, CHEK1, E2F3, MSX1, RAN, MAD2L1 |
| BP | GO:0008015~blood circulation | 5 | 0.935 | 0.012 | CXCL10, CXCL12, EDN3, STAT1, CHD7 |
| BP | GO:0051973~positive regulation of telomerase activity | 5 | 0.935 | 0.012 | CCT2, HSP90AB1, DKC1, MYC, AURKB |
| MF | GO:0005179~hormone activity | 9 | 1.682 | 0.013 | PYY, REG3A, EDN3, SST, STC2, GCG, VIP, GUCA2A, AGT |
| MF | GO:0042834~peptidoglycan binding | 4 | 0.748 | 0.013 | REG3A, REG1B, REG1A, ZG16 |
| MF | GO:0044183~protein binding involved in protein folding | 6 | 1.121 | 0.013 | CCT3, CCT6A, CCT2, HSP90AB1, HSPA2, CCT7 |
| BP | GO:0045926~negative regulation of growth | 4 | 0.748 | 0.013 | MT1M, MT1F, MT1G, MT1E |
| BP | GO:2000406~positive regulation of T cell migration | 4 | 0.748 | 0.013 | CXCL10, CXCL12, CCL21, CCL20 |
| BP | GO:0097294~'de novo' XMP biosynthetic process | 3 | 0.561 | 0.014 | ATIC, PPAT, PAICS |
| KEGG_PATHWAY | hsa00480:Glutathione metabolism | 7 | 1.308 | 0.015 | GGCT, GPX2, RRM2, GPX3, ANPEP, ODC1, SRM |
| CC | GO:0044297~cell body | 7 | 1.308 | 0.015 | CCT3, SLC12A2, CCT2, TUBB, DPYSL3, CCT7, ACTG2 |
| BP | GO:0019229~regulation of vasoconstriction | 4 | 0.748 | 0.015 | EDN3, AGTR1, ATP1A2, AGT |
| MF | GO:0004252~serine-type endopeptidase activity | 12 | 2.243 | 0.016 | MMP12, CFD, MMP11, PCID2, MMP7, PLAU, MMP3, GZMB, CTSG, MMP9, CFB, C2 |
| CC | GO:0005635~nuclear envelope | 12 | 2.243 | 0.016 | RNF43, RANBP1, CENPF, NUP205, GOLT1A, MYC, CSE1L, RANGAP1, LMNB2, RAN, HTATIP2, NUP37 |
| BP | GO:0016055~Wnt signaling pathway | 12 | 2.243 | 0.016 | RNF43, SFRP1, SFRP2, CCND1, LEF1, DACT3, AXIN2, ZBTB33, APCDD1, FERMT2, NKD1, NKD2 |
| BP | GO:0071260~cellular response to mechanical stimulus | 7 | 1.308 | 0.016 | COL1A1, ITGA2, CHEK1, SLC2A1, ATP1A2, SOX9, AGT |
| BP | GO:0060412~ventricular septum morphogenesis | 5 | 0.935 | 0.016 | CITED2, SLIT2, PROX1, SOX4, FGFRL1 |
| BP | GO:0000070~mitotic sister chromatid segregation | 5 | 0.935 | 0.016 | KIFC1, NUSAP1, CDCA8, RAN, MAD2L1 |
| BP | GO:0071276~cellular response to cadmium ion | 5 | 0.935 | 0.016 | MT1M, MT1F, MT1G, MMP9, MT1E |
| BP | GO:0031100~animal organ regeneration | 5 | 0.935 | 0.016 | CCNA2, CXCL12, ATIC, ANGPT2, CDK1 |
| BP | GO:0042594~response to starvation | 5 | 0.935 | 0.016 | PDK4, GPT, HMGCS2, PPARGC1A, LRP11 |
| CC | GO:0030055~cell-substrate junction | 3 | 0.561 | 0.017 | FERMT1, PGM5, FERMT2 |
| BP | GO:0045600~positive regulation of fat cell differentiation | 6 | 1.121 | 0.017 | SFRP1, CEBPB, SFRP2, ZBTB16, LMO3, AXIN2 |
| MF | GO:0030515~snoRNA binding | 4 | 0.748 | 0.017 | NOP56, HEATR1, DDX21, BYSL |
| MF | GO:0017116~single-stranded DNA-dependent ATP-dependent DNA helicase activity | 4 | 0.748 | 0.017 | RFC4, MCM3, MCM4, MCM2 |
| CC | GO:0000794~condensed nuclear chromosome | 5 | 0.935 | 0.017 | SUV39H1, CHEK1, RRS1, NCAPG, NCAPD2 |
| CC | GO:0042470~melanosome | 8 | 1.495 | 0.017 | HSP90AB1, AHCY, FASN, SLC2A1, GGH, MYH11, SND1, RAN |
| BP | GO:0007517~muscle organ development | 8 | 1.495 | 0.018 | CXCL10, CENPF, TAGLN, SMTN, FHL1, MSX1, JPH1, CRYAB |
| BP | GO:0009612~response to mechanical stimulus | 6 | 1.121 | 0.018 | COL1A1, ANGPT2, CITED2, STAT1, FOSB, LRP11 |
| BP | GO:0006271~DNA strand elongation involved in DNA replication | 3 | 0.561 | 0.018 | RFC4, MCM3, MCM4 |
| BP | GO:0060087~relaxation of vascular smooth muscle | 3 | 0.561 | 0.018 | RGS2, ADORA2B, KCNMA1 |
| BP | GO:0044208~'de novo' AMP biosynthetic process | 3 | 0.561 | 0.018 | ATIC, PPAT, PAICS |
| CC | GO:0005697~telomerase holoenzyme complex | 4 | 0.748 | 0.018 | DKC1, NHP2, NAT10, SNRPB |
| BP | GO:0007179~transforming growth factor beta receptor signaling pathway | 8 | 1.495 | 0.019 | CLDN5, COL3A1, COL1A2, HPGD, CITED2, BAMBI, GDF15, FERMT2 |
| BP | GO:0032869~cellular response to insulin stimulus | 8 | 1.495 | 0.019 | GHR, STAT1, CDK4, MYC, TRIB3, IRS2, GPT, HMGCS2 |
| CC | GO:0005923~bicellular tight junction | 9 | 1.682 | 0.019 | CLDN5, CCND1, CDK4, CLDN8, CLDN23, CLDN2, ECT2, CLDN1, JAM2 |
| BP | GO:0000086~G2/M transition of mitotic cell cycle | 6 | 1.121 | 0.019 | CCNA2, MELK, CHEK1, CDK1, CDC25B, AURKA |
| BP | GO:0007584~response to nutrient | 6 | 1.121 | 0.019 | TMIGD1, SFRP2, STAT1, SST, LIPG, HMGCS2 |
| MF | GO:0042379~chemokine receptor binding | 3 | 0.561 | 0.019 | CXCL12, CCL21, CCL19 |
| KEGG_PATHWAY | hsa04670:Leukocyte transendothelial migration | 10 | 1.869 | 0.019 | CLDN5, CXCL12, CLDN8, CLDN23, THY1, CLDN2, CLDN1, MYL9, MMP9, JAM2 |
| MF | GO:0005198~structural molecule activity | 12 | 2.243 | 0.019 | CLDN5, CLDN8, TUBB, EPB41L3, CLDN23, PGM5, OLFM4, CLDN2, CRYAB, CLDN1, SNTB1, PRPH |
| BP | GO:0046718~viral entry into host cell | 8 | 1.495 | 0.019 | ANPEP, ITGA2, CDK1, AGTR1, CLDN1, SLC7A1, LDLR, CD55 |
| BP | GO:0061077~chaperone-mediated protein folding | 5 | 0.935 | 0.020 | CCT3, CCT6A, CCT2, TRAP1, CCT7 |
| BP | GO:0001525~angiogenesis | 14 | 2.617 | 0.020 | ANGPT2, TNFRSF12A, CAV1, THY1, ESM1, MEIS1, SRPX2, CALD1, ANPEP, TGFBI, SCG2, EPHB4, EPHB3, HTATIP2 |
| CC | GO:0005730~nucleolus | 50 | 9.346 | 0.020 | TOP2A, FEN1, SUV39H1, CHD7, HJURP, NAT10, RGS2, MYC, ENC1, NUSAP1, CHP2, CA9, RRP12, PHLDA1, DNTTIP1, AEN, ZBTB33, ETV4, GTPBP4, GNL3, DKC1, NHP2, CCDC86, ZNF593, SF3B3, HEATR1, NOLC1, DDX21, CDCA8, RPF2, SELENBP1, EXOSC7, EXOSC5, RRS1, BRIX1, E2F5, LYAR, NOP56, CENPW, STAT1, RCC2, PA2G4, NPM3, BYSL, MAD2L2, RSL1D1, SCD, CDK4, UBE2T, RAN |
| KEGG_PATHWAY | hsa00910:Nitrogen metabolism | 4 | 0.748 | 0.021 | CA1, CA2, CA4, CA9 |
| CC | GO:0005732~small nucleolar ribonucleoprotein complex | 3 | 0.561 | 0.021 | NOP56, NHP2, SNRPB |
| MF | GO:0001618~virus receptor activity | 7 | 1.308 | 0.021 | ANPEP, ITGA2, CDK1, CLDN1, SLC7A1, LDLR, CD55 |
| CC | GO:0000785~chromatin | 39 | 7.290 | 0.021 | CEBPB, CITED2, UHRF1, CDCA5, CHD7, ASCL2, ETS2, RAD51AP1, FOXQ1, SPIB, MYC, CHEK1, ENC1, OIP5, E2F3, SOX9, MSX1, E2F5, PPARGC1A, PITX1, NKX2-3, SOX4, HES6, SMARCC1, STAT1, ACTL6A, ZBTB33, PROX1, ETV4, MAD2L2, MEIS1, CDK4, KLF9, NFE2L3, TIMELESS, FOSB, HOXB8, MCM2, ASF1B |
| CC | GO:0000922~spindle pole | 9 | 1.682 | 0.021 | CDC20, TPX2, CENPF, CKAP2L, PRC1, NEK6, CKAP2, CDC25B, MAD2L1 |
| BP | GO:0046777~protein autophosphorylation | 11 | 2.056 | 0.022 | MELK, IRAK1, NEK6, CAD, TTK, THY1, EPHB4, AURKB, EPHB3, AURKA, HTATIP2 |
| BP | GO:0002053~positive regulation of mesenchymal cell proliferation | 4 | 0.748 | 0.022 | STAT1, MYC, IRS2, SOX9 |
| BP | GO:1902570~protein localization to nucleolus | 3 | 0.561 | 0.023 | RRS1, RPF2, RAN |
| BP | GO:0030728~ovulation | 3 | 0.561 | 0.023 | RGS2, HPGD, MYC |
| BP | GO:0035360~positive regulation of peroxisome proliferator activated receptor signaling pathway | 3 | 0.561 | 0.023 | PTGIS, CITED2, LMO3 |
| BP | GO:1990481~mRNA pseudouridine synthesis | 3 | 0.561 | 0.023 | RPUSD4, DKC1, PUS7 |
| BP | GO:2000669~negative regulation of dendritic cell apoptotic process | 3 | 0.561 | 0.023 | CXCL12, CCL21, CCL19 |
| BP | GO:0051248~negative regulation of protein metabolic process | 3 | 0.561 | 0.023 | HSP90AB1, MIF, LDLR |
| BP | GO:0006177~GMP biosynthetic process | 3 | 0.561 | 0.023 | ATIC, PPAT, PAICS |
| CC | GO:0031225~anchored component of membrane | 8 | 1.495 | 0.023 | CEACAM7, CA4, ITLN1, DPEP1, THY1, LY6G6D, CD55, LY6E |
| KEGG_PATHWAY | hsa05160:Hepatitis C | 12 | 2.243 | 0.023 | CLDN5, CXCL10, CCND1, STAT1, CDK4, MYC, CLDN8, CLDN23, E2F3, CLDN2, CLDN1, LDLR |
| BP | GO:0030574~collagen catabolic process | 5 | 0.935 | 0.023 | MMP12, MMP11, MMP7, MMP3, MMP9 |
| BP | GO:1901796~regulation of signal transduction by p53 class mediator | 5 | 0.935 | 0.023 | CHEK1, RRS1, RPF2, AURKB, AURKA |
| BP | GO:0030335~positive regulation of cell migration | 14 | 2.617 | 0.024 | SEMA6A, MMP7, SEMA4D, CAV1, LEF1, FAM107A, IRS2, CLDN1, MMP9, MYLK, COL1A1, CXCL12, PLAU, FERMT2 |
| KEGG_PATHWAY | hsa04933:AGE-RAGE signaling pathway in diabetic complications | 9 | 1.682 | 0.025 | COL1A1, COL3A1, COL1A2, CCND1, COL4A1, STAT1, CDK4, AGTR1, AGT |
| BP | GO:0008380~RNA splicing | 12 | 2.243 | 0.025 | RPUSD4, EFTUD2, PRPF4, SYNCRIP, PPIL1, SF3B3, PRMT1, TTF2, SNRPF, PUS7, PPARGC1A, SNRPB |
| BP | GO:0007080~mitotic metaphase plate congression | 5 | 0.935 | 0.025 | KIFC1, CDCA5, RRS1, CDCA8, KIF2C |
| BP | GO:0046597~negative regulation of viral entry into host cell | 4 | 0.748 | 0.025 | IFITM3, IFITM1, IFITM2, LY6E |
| BP | GO:0006883~cellular sodium ion homeostasis | 4 | 0.748 | 0.025 | SLC12A2, SCNN1B, ATP1A2, AGT |
| BP | GO:0006457~protein folding | 11 | 2.056 | 0.026 | CCT3, CCT6A, CCT2, TRAP1, PPIL1, HSPH1, HSP90AB1, GRPEL2, CCT7, CRYAB, PDRG1 |
| CC | GO:0009925~basal plasma membrane | 6 | 1.121 | 0.027 | SLC5A6, SLC12A2, SLC7A5, SLCO1B3, TACSTD2, SLC7A1 |
| BP | GO:0034504~protein localization to nucleus | 5 | 0.935 | 0.027 | COL1A1, ZBTB16, SOX9, MSX1, BMP7 |
| BP | GO:0042307~positive regulation of protein import into nucleus | 5 | 0.935 | 0.027 | HSP90AB1, CDK1, CHP2, ECT2, RAN |
| BP | GO:0071456~cellular response to hypoxia | 9 | 1.682 | 0.027 | CCNA2, SFRP1, PTGIS, IRAK1, MYC, KCNMB1, STC2, SUV39H1, BMP7 |
| KEGG_PATHWAY | hsa04972:Pancreatic secretion | 9 | 1.682 | 0.028 | SLC12A2, CA2, KCNMA1, ADCY3, ATP1A2, CEL, SLC26A3, SLC4A4, CLCA4 |
| KEGG_PATHWAY | hsa04914:Progesterone-mediated oocyte maturation | 9 | 1.682 | 0.028 | CCNA2, MAD2L2, HSP90AB1, CDK1, ADCY3, BUB1, CDC25B, MAD2L1, AURKA |
| BP | GO:0035999~tetrahydrofolate interconversion | 3 | 0.561 | 0.028 | MTHFD1L, SHMT2, MTHFD2 |
| BP | GO:0071294~cellular response to zinc ion | 4 | 0.748 | 0.028 | MT1M, MT1F, MT1G, MT1E |
| MF | GO:0003678~DNA helicase activity | 6 | 1.121 | 0.028 | CHD7, MCM3, MCM4, MCM6, GTF2F2, MCM2 |
| CC | GO:0034774~secretory granule lumen | 8 | 1.495 | 0.029 | CFD, PRDX4, HSP90AB1, GCG, S100P, MIF, S100A9, S100A11 |
| KEGG_PATHWAY | hsa04936:Alcoholic liver disease | 11 | 2.056 | 0.029 | CCND1, IRAK1, ADH1C, ADH1B, SCD, FASN, LEF1, CXCL1, CXCL2, PPARGC1A, C2 |
| CC | GO:0035578~azurophil granule lumen | 7 | 1.308 | 0.029 | CCT2, ACLY, TUBB, GGH, CTSG, PA2G4, GLA |
| MF | GO:0008097~5S rRNA binding | 3 | 0.561 | 0.030 | GTF3A, RRS1, RPF2 |
| BP | GO:0043065~positive regulation of apoptotic process | 16 | 2.991 | 0.030 | TOP2A, TNFRSF12A, HPGD, RIPK2, ZBTB16, PDCD5, BMP7, MMP9, SFRP1, MELK, SFRP2, KCNMA1, SLIT2, ECT2, PHLDA1, SOX4 |
| MF | GO:0004867~serine-type endopeptidase inhibitor activity | 8 | 1.495 | 0.030 | SPINK4, SERPINE2, COL7A1, SLCO1B3, SERPINH1, PI3, SERPINB5, AGT |
| BP | GO:0030154~cell differentiation | 28 | 5.234 | 0.031 | SERPINE2, SUV39H1, FHL1, BZW2, ETS2, FOXQ1, CDC20, SPIB, RNF114, ANPEP, FLNC, ECT2, NKX2-3, SOX4, HTATIP2, HES6, SEMA6A, TNFRSF12A, TESC, CAV1, CEL, ETV4, SFRP1, CENPF, SFRP2, MGP, MAL, ASF1B |
| BP | GO:0006956~complement activation | 4 | 0.748 | 0.031 | CFD, C7, CFB, C2 |
| BP | GO:0007346~regulation of mitotic cell cycle | 6 | 1.121 | 0.031 | CDC20, MYC, NEK6, CKS2, DLGAP5, CKS1B |
| BP | GO:0043407~negative regulation of MAP kinase activity | 5 | 0.935 | 0.032 | UCHL1, RGS2, CAV1, BMP7, AGT |
| BP | GO:0071222~cellular response to lipopolysaccharide | 11 | 2.056 | 0.032 | CXCL10, SLC7A5, CEBPB, CXCL9, IRAK1, DEFA6, CDK4, CTSG, CXCL1, HMGCS2, CXCL2 |
| MF | GO:0005178~integrin binding | 10 | 1.869 | 0.032 | FERMT1, COL3A1, ESM1, CXCL12, SFRP2, ITGA2, TGFBI, THY1, FERMT2, JAM2 |
| KEGG_PATHWAY | hsa00100:Steroid biosynthesis | 4 | 0.748 | 0.032 | SQLE, NSDHL, CEL, DHCR7 |
| KEGG_PATHWAY | hsa00670:One carbon pool by folate | 4 | 0.748 | 0.032 | ATIC, MTHFD1L, SHMT2, MTHFD2 |
| MF | GO:0008017~microtubule binding | 14 | 2.617 | 0.033 | RCC2, POLB, REEP1, TPX2, CENPF, KIFC1, PRC1, KIF4A, NUSAP1, KIF2C, KIF20A, CRYAB, S100A9, DLGAP5 |
| BP | GO:0035455~response to interferon-alpha | 3 | 0.561 | 0.033 | IFITM3, IFITM1, IFITM2 |
| BP | GO:0071492~cellular response to UV-A | 3 | 0.561 | 0.033 | MMP3, TIMP1, MMP9 |
| BP | GO:0030644~cellular chloride ion homeostasis | 3 | 0.561 | 0.033 | SLC12A2, TBXAS1, CKB |
| BP | GO:0000076~DNA replication checkpoint | 3 | 0.561 | 0.033 | CDT1, CDC45, TIMELESS |
| BP | GO:0071313~cellular response to caffeine | 3 | 0.561 | 0.033 | CHEK1, CASQ2, RYR3 |
| CC | GO:0005643~nuclear pore | 7 | 1.308 | 0.034 | RANBP1, NUP205, NXT1, RANGAP1, NUP62CL, RAN, NUP37 |
| BP | GO:0071407~cellular response to organic cyclic compound | 5 | 0.935 | 0.034 | CEBPB, HSP90AB1, STAT1, CDK1, AXIN2 |
| MF | GO:0048027~mRNA 5'-UTR binding | 4 | 0.748 | 0.034 | SYNCRIP, RSL1D1, SHMT2, GNL3 |
| MF | GO:0005539~glycosaminoglycan binding | 4 | 0.748 | 0.034 | VCAN, SERPINE2, BGN, SULF1 |
| BP | GO:0034341~response to interferon-gamma | 4 | 0.748 | 0.034 | IFITM3, IFITM1, IFITM2, STAT1 |
| MF | GO:0015269~calcium-activated potassium channel activity | 3 | 0.561 | 0.036 | KCNMB1, KCNMA1, KCNN4 |
| MF | GO:0019911~structural constituent of myelin sheath | 3 | 0.561 | 0.036 | CMTM8, MAL, GPM6B |
| MF | GO:0019899~enzyme binding | 18 | 3.364 | 0.036 | TOP2A, BCHE, TRAP1, HSP90AB1, PRMT1, STAT1, CAV1, CAD, HSPA2, AXIN2, PUS7, POLB, CDC20, CCND1, STC2, DUOXA2, SLC27A2, MCM2 |
| CC | GO:0072687~meiotic spindle | 3 | 0.561 | 0.036 | ASPM, HSPA2, AURKA |
| BP | GO:0033138~positive regulation of peptidyl-serine phosphorylation | 7 | 1.308 | 0.037 | MAD2L2, SFRP2, HSP90AB1, RIPK2, CAV1, GCG, MIF |
| CC | GO:0000781~chromosome, telomeric region | 10 | 1.869 | 0.037 | RAD51AP1, FEN1, CHEK1, NHP2, CDK1, MCM3, NAT10, MCM4, MCM6, MCM2 |
| BP | GO:0098609~cell-cell adhesion | 11 | 2.056 | 0.037 | TMEM47, TMIGD1, SRPX2, CDH3, ITGA2, CDH11, SOX9, THY1, CLDN2, S100A11, JAM2 |
| BP | GO:0071280~cellular response to copper ion | 4 | 0.748 | 0.038 | MT1M, MT1F, MT1G, MT1E |
| BP | GO:0035116~embryonic hindlimb morphogenesis | 4 | 0.748 | 0.038 | ZBTB16, CHD7, MSX1, PITX1 |
| CC | GO:0071005~U2-type precatalytic spliceosome | 5 | 0.935 | 0.039 | EFTUD2, PRPF4, SF3B3, SNRPF, SNRPB |
| MF | GO:0003723~RNA binding | 53 | 9.907 | 0.039 | TOP2A, HSP90AB1, NAT10, EFTUD2, SYNCRIP, NUSAP1, RRP12, CCT3, PCID2, PRMT1, DDX10, RANGAP1, PPRC1, PUS7, GTPBP4, SND1, GNL3, RPUSD4, DKC1, FSCN1, NHP2, CCDC86, ZNF239, SNRPF, SNRPB, DNMT1, HEATR1, NOLC1, DDX21, RPF2, RAD51AP1, EXOSC7, STIP1, EXOSC5, RRS1, SERPINH1, BRIX1, PPARGC1A, LYAR, NOP56, TRAP1, YTHDF1, CPSF3, RCC2, PA2G4, NPM3, BYSL, CCT6A, RSL1D1, FASN, SSBP1, RAN, EIF3B |
| BP | GO:0001822~kidney development | 8 | 1.495 | 0.039 | CENPF, HPGD, ODC1, AGTR1, HMGCS2, PROX1, SULF1, AGT |
| BP | GO:0034501~protein localization to kinetochore | 3 | 0.561 | 0.039 | CDK1, TTK, AURKB |
| BP | GO:0048251~elastic fiber assembly | 3 | 0.561 | 0.039 | MFAP4, COL3A1, MYH11 |
| BP | GO:0038166~angiotensin-activated signaling pathway | 3 | 0.561 | 0.039 | CA2, AGTR1, AGT |
| BP | GO:0051983~regulation of chromosome segregation | 3 | 0.561 | 0.039 | KIF2C, BUB1, AURKB |
| KEGG_PATHWAY | hsa04310:Wnt signaling pathway | 12 | 2.243 | 0.040 | RNF43, SFRP1, SFRP2, MMP7, CCND1, BAMBI, MYC, LEF1, AXIN2, APCDD1, NKD1, NKD2 |
| CC | GO:0101031~chaperone complex | 4 | 0.748 | 0.041 | STIP1, HSPB8, PSMG1, PDRG1 |
| CC | GO:0071007~U2-type catalytic step 2 spliceosome | 4 | 0.748 | 0.041 | EFTUD2, PPIL1, SNRPF, SNRPB |
| KEGG_PATHWAY | hsa05202:Transcriptional misregulation in cancer | 13 | 2.430 | 0.041 | CEBPB, DEFA6, HPGD, ZBTB16, MMP3, GZMB, ETV4, MMP9, CCNA2, MEIS1, PLAU, MYC, TSPAN7 |
| BP | GO:0007568~aging | 9 | 1.682 | 0.041 | SLC12A2, POLB, IRAK1, RPN2, KCNMB1, TIMP1, CLDN1, AGT, AURKB |
| CC | GO:0016328~lateral plasma membrane | 6 | 1.121 | 0.041 | SLC12A2, CLDN5, GPSM2, TACSTD2, CLDN1, NKD2 |
| CC | GO:0030133~transport vesicle | 7 | 1.308 | 0.042 | CHGA, DEFA6, SSPN, BGN, KDELR3, CD55, FGFRL1 |
| BP | GO:0050821~protein stabilization | 12 | 2.243 | 0.042 | CCT3, CCT6A, CCT2, HSP90AB1, TESC, MSX1, CCT7, CRYAB, PPARGC1A, SOX4, GTPBP4, PDRG1 |
| MF | GO:0005092~GDP-dissociation inhibitor activity | 3 | 0.561 | 0.042 | GPSM2, RANBP1, SH3BP4 |
| CC | GO:0010369~chromocenter | 3 | 0.561 | 0.042 | CDCA8, OIP5, AURKB |
| KEGG_PATHWAY | hsa05222:Small cell lung cancer | 8 | 1.495 | 0.044 | CCND1, COL4A1, CDK4, MYC, ITGA2, CKS2, E2F3, CKS1B |
| CC | GO:0005912~adherens junction | 10 | 1.869 | 0.044 | TMEM47, SYNM, PDLIM3, CDH3, CDCA3, CDH11, NEXN, PGM5, FERMT2, S100A11 |
| MF | GO:0005509~calcium ion binding | 30 | 5.607 | 0.044 | ITLN1, NKD1, RYR3, NKD2, SCGN, CLEC3B, CDH3, NCS1, CHP2, SLIT2, SLC25A23, LDLR, S100A11, PCID2, TESC, SULF1, MMP12, PCP4, VCAN, MELK, VSNL1, MGP, CDH11, CASQ2, SPARCL1, S100P, TKT, S100A9, MYL9, MATN2 |
| CC | GO:0005856~cytoskeleton | 22 | 4.112 | 0.045 | CCT3, TMOD1, TAGLN, FNBP1, RIPK2, TPM2, TUBB, LMOD1, ACTG2, CNN1, FERMT1, SMTN, CALD1, PRC1, KIF4A, EPB41L3, ENC1, FSCN1, FLNC, S100A9, FERMT2, SNTB1 |
| BP | GO:0043589~skin morphogenesis | 3 | 0.561 | 0.046 | COL1A1, COL1A2, ITGA2 |
| BP | GO:0001522~pseudouridine synthesis | 3 | 0.561 | 0.046 | RPUSD4, DKC1, PUS7 |
| BP | GO:0003014~renal system process | 3 | 0.561 | 0.046 | SCNN1B, SLC25A23, AGT |
| BP | GO:0006690~icosanoid metabolic process | 3 | 0.561 | 0.046 | PTGIS, CYP2S1, TBXAS1 |
| BP | GO:0050732~negative regulation of peptidyl-tyrosine phosphorylation | 3 | 0.561 | 0.046 | SFRP1, SFRP2, SEMA4D |
| KEGG_PATHWAY | hsa04614:Renin-angiotensin system | 4 | 0.748 | 0.046 | ANPEP, AGTR1, CTSG, AGT |
| KEGG_PATHWAY | hsa04964:Proximal tubule bicarbonate reclamation | 4 | 0.748 | 0.046 | CA2, CA4, ATP1A2, SLC4A4 |
| KEGG_PATHWAY | hsa04970:Salivary secretion | 8 | 1.495 | 0.046 | SLC12A2, CST1, KCNMA1, ADCY3, ATP1A2, KCNN4, BEST2, RYR3 |
| BP | GO:0009408~response to heat | 5 | 0.935 | 0.047 | CXCL12, SST, HSPA2, CRYAB, LRP11 |
| BP | GO:0006935~chemotaxis | 8 | 1.495 | 0.047 | CXCL10, CMTM8, CXCL9, CXCL12, PLAU, CCL20, CXCL1, CXCL2 |
| CC | GO:0031616~spindle pole centrosome | 3 | 0.561 | 0.049 | DLGAP5, AURKB, AURKA |
| CC | GO:1990023~mitotic spindle midzone | 3 | 0.561 | 0.049 | PRC1, RCC2, AURKB |
| MF | GO:0009982~pseudouridine synthase activity | 3 | 0.561 | 0.049 | RPUSD4, DKC1, PUS7 |
| BP | GO:0007588~excretion | 4 | 0.748 | 0.049 | ADORA2B, SCNN1B, GUCA2B, SLC26A3 |
| BP | GO:0010033~response to organic substance | 4 | 0.748 | 0.049 | SQLE, CDK4, S100P, TIMP1 |
| BP | GO:0071346~cellular response to interferon-gamma | 7 | 1.308 | 0.049 | SYNCRIP, CCL21, STAT1, CCL20, MYC, CCL19, CLDN1 |

**Supplementary Table 7.** Results of GSEA analysis exploring the eosinophil-related shared pathways involving PPP1R14A in colon cancer.

| **Description** | **ES** | **NES** | **NOM p-val** | **FDR q-val** | **FWER p-val** | |
| --- | --- | --- | --- | --- | --- | --- |
| PPP1R14A-LOW EXPRESSION GROUP- Colon cancer | | | | | | |
| GALACTOSE METABOLISM | -0.746 | -2.077 | 0 | 0.106 | 0.033 | |
| ARACHIDONIC ACID METABOLISM | -0.577 | -1.968 | 0.002 | 0.180 | 0.086 | |
| CHEMOKINE SIGNALING PATHWAY | -0.567 | -1.912 | 0.018 | 0.226 | 0.136 | |
| NATURAL KILLER CELL MEDIATED CYTOTOXICITY | -0.554 | -1.912 | 0.018 | 0.170 | 0.137 | |
| NOTCH SIGNALING PATHWAY | -0.659 | -1.895 | 0.010 | 0.158 | 0.152 | |
| CYTOKINE CYTOKINE RECEPTOR INTERACTION | -0.525 | -1.875 | 0.016 | 0.159 | 0.170 |  |
| GLYCOSAMINOGLYCAN DEGRADATION | -0.680 | -1.873 | 0.006 | 0.139 | 0.174 |  |
| VEGF SIGNALING PATHWAY | -0.536 | -1.838 | 0.014 | 0.168 | 0.209 |  |
| LEISHMANIA INFECTION | -0.629 | -1.821 | 0.034 | 0.169 | 0.229 |  |
| LYSOSOME | -0.590 | -1.819 | 0.028 | 0.154 | 0.23 |  |
| ASTHMA | -0.706 | -1.789 | 0.030 | 0.181 | 0.275 |  |
| VASOPRESSIN REGULATED WATER REABSORPTION | -0.586 | -1.787 | 0.018 | 0.167 | 0.276 |  |
| FRUCTOSE AND MANNOSE METABOLISM | -0.613 | -1.778 | 0.018 | 0.166 | 0.287 |  |
| T CELL RECEPTOR SIGNALING PATHWAY | -0.543 | -1.769 | 0.039 | 0.165 | 0.298 |  |
| B CELL RECEPTOR SIGNALING PATHWAY | -0.562 | -1.764 | 0.029 | 0.160 | 0.306 |  |
| FC EPSILON RI SIGNALING PATHWAY | -0.516 | -1.764 | 0.020 | 0.150 | 0.306 |  |
| BASAL CELL CARCINOMA | -0.557 | -1.755 | 0.029 | 0.150 | 0.316 |  |
| COMPLEMENT AND COAGULATION CASCADES | -0.559 | -1.751 | 0.026 | 0.147 | 0.322 |  |
| PANTOTHENATE AND COA BIOSYNTHESIS | -0.622 | -1.748 | 0.019 | 0.143 | 0.33 |  |
| INTESTINAL IMMUNE NETWORK FOR IGA PRODUCTION | -0.672 | -1.744 | 0.039 | 0.139 | 0.337 |  |
| OTHER GLYCAN DEGRADATION | -0.721 | -1.742 | 0.018 | 0.134 | 0.339 |  |
| FC GAMMA R MEDIATED PHAGOCYTOSIS | -0.538 | -1.738 | 0.038 | 0.132 | 0.348 |  |
| AUTOIMMUNE THYROID DISEASE | -0.594 | -1.729 | 0.038 | 0.136 | 0.369 |  |
| GRAFT VERSUS HOST DISEASE | -0.722 | -1.725 | 0.037 | 0.133 | 0.375 |  |
| GLYCOSPHINGOLIPID BIOSYNTHESIS GANGLIO SERIES | -0.624 | -1.718 | 0.014 | 0.135 | 0.385 |  |
| GLYCOSAMINOGLYCAN BIOSYNTHESIS CHONDROITIN SULFATE | -0.671 | -1.717 | 0.033 | 0.131 | 0.389 |  |
| TOLL LIKE RECEPTOR SIGNALING PATHWAY | -0.496 | -1.706 | 0.041 | 0.131 | 0.407 |  |
| HEMATOPOIETIC CELL LINEAGE | -0.575 | -1.704 | 0.047 | 0.128 | 0.412 |  |
| WNT SIGNALING PATHWAY | -0.478 | -1.703 | 0.029 | 0.125 | 0.413 |  |
| HEDGEHOG SIGNALING PATHWAY | -0.524 | -1.699 | 0.029 | 0.123 | 0.417 |  |
| ACUTE MYELOID LEUKEMIA | -0.556 | -1.690 | 0.046 | 0.126 | 0.428 |  |
| PATHWAYS IN CANCER | -0.470 | -1.680 | 0.048 | 0.126 | 0.438 |  |
| MTOR SIGNALING PATHWAY | -0.503 | -1.666 | 0.032 | 0.130 | 0.459 |  |
| JAK STAT SIGNALING PATHWAY | -0.446 | -1.646 | 0.045 | 0.133 | 0.483 |  |
| ABC TRANSPORTERS | -0.491 | -1.629 | 0.018 | 0.137 | 0.508 |  |
| GLYCEROPHOSPHOLIPID METABOLISM | -0.448 | -1.566 | 0.045 | 0.166 | 0.584 |  |
| MATURITY ONSET DIABETES OF THE YOUNG | -0.600 | -1.563 | 0.034 | 0.166 | 0.591 |  |
| ETHER LIPID METABOLISM | -0.497 | -1.552 | 0.039 | 0.165 | 0.605 |  |
| PRIMARY BILE ACID BIOSYNTHESIS | -0.542 | -1.533 | 0.032 | 0.169 | 0.621 |  |

**Supplementary Table 8.** Results of GSEA analysis exploring the eosinophil-related shared pathways involving PPP1R14A in asthma.

| **Description** | **ES** | **NES** | **NOM p-val** | **FDR q-val** | **FWER p-val** |
| --- | --- | --- | --- | --- | --- |
| PPP1R14A-LOW EXPRESSION GROUP-Asthma | | | | | |
| LYSOSOME | -0.509 | -1.591 | 0.058 | 0.239 | 0.624 |
| PROTEIN EXPORT | -0.711 | -1.591 | 0.035 | 0.213 | 0.626 |
| PARKINSONS DISEASE | -0.551 | -1.590 | 0.041 | 0.194 | 0.63 |
| OXIDATIVE PHOSPHORYLATION | -0.549 | -1.589 | 0.047 | 0.178 | 0.632 |
| BUTANOATE METABOLISM | -0.518 | -1.559 | 0.038 | 0.208 | 0.685 |
| N GLYCAN BIOSYNTHESIS | -0.595 | -1.529 | 0.043 | 0.245 | 0.751 |
| DNA REPLICATION | -0.537 | -1.527 | 0.030 | 0.233 | 0.759 |
| GLYCOLYSIS GLUCONEOGENESIS | -0.449 | -1.516 | 0.036 | 0.236 | 0.779 |
| PYRIMIDINE METABOLISM | -0.433 | -1.487 | 0.056 | 0.208 | 0.827 |
| PENTOSE PHOSPHATE PATHWAY | -0.515 | -1.439 | 0.055 | 0.239 | 0.903 |
