# Supplementary figures and images for: Role of eosinophil counts in mediating the association between asthma and colon cancer
Source: Clin Transl Allergy. 2024 Dec 10;14(12):e70012. doi: 10.1002/clt2.70012 (PMC11632118; doi:10.1002/clt2.70012)

**A**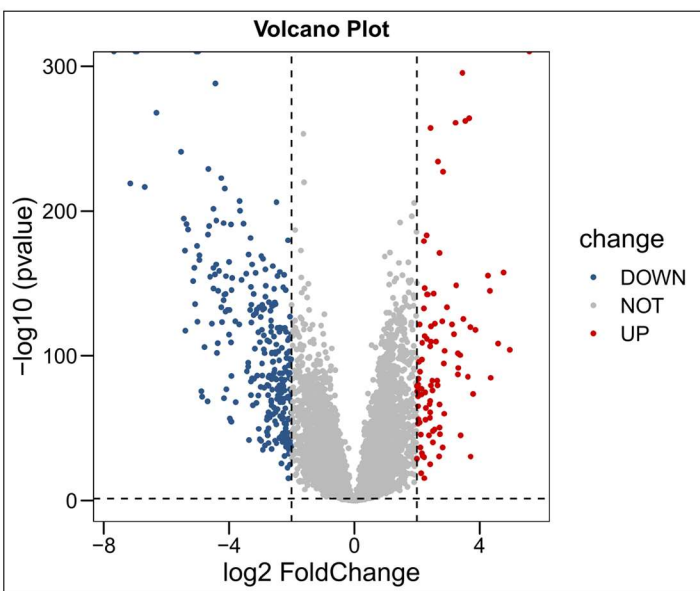**B**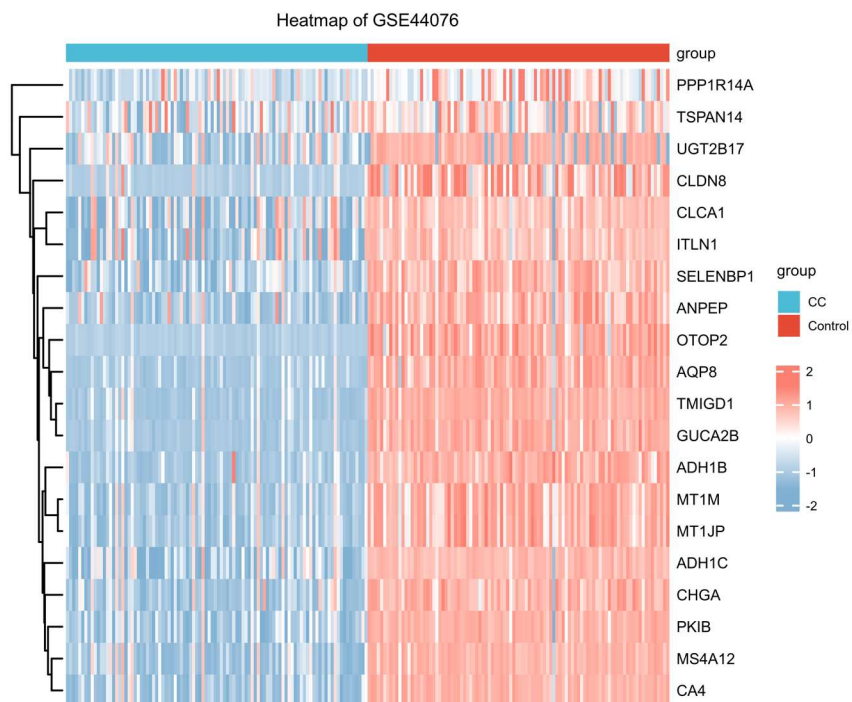**C**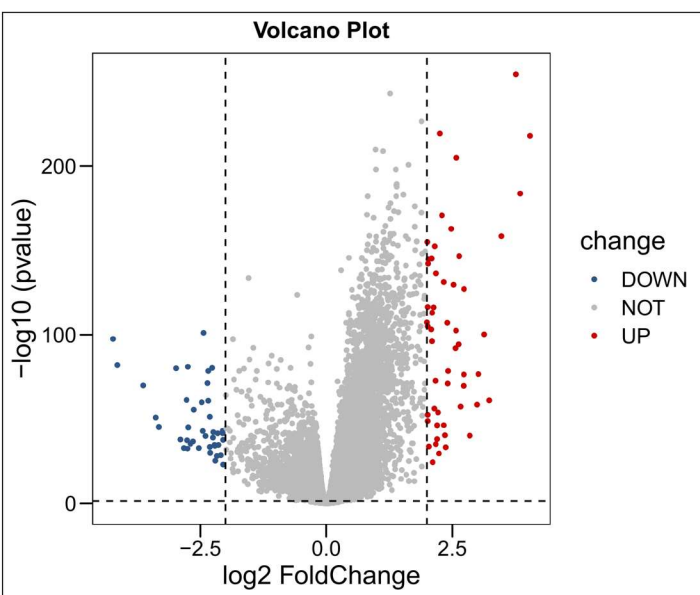**D**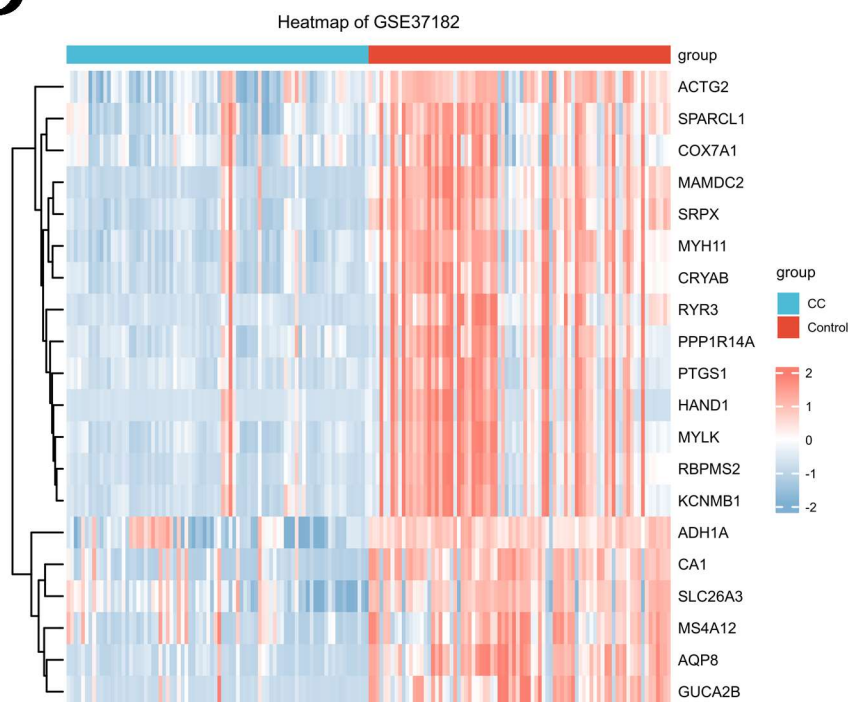**E**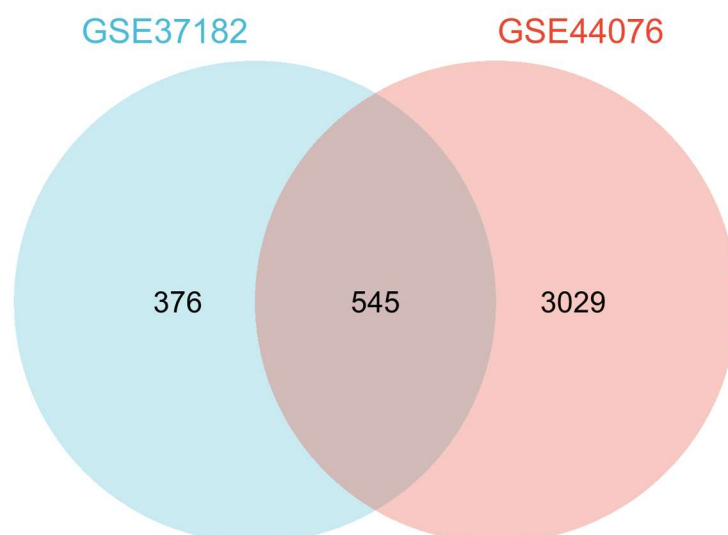

Supplement: Supplementary file 3 — Figure S1 [file CLT2-14-e70012-s001.pdf]

A

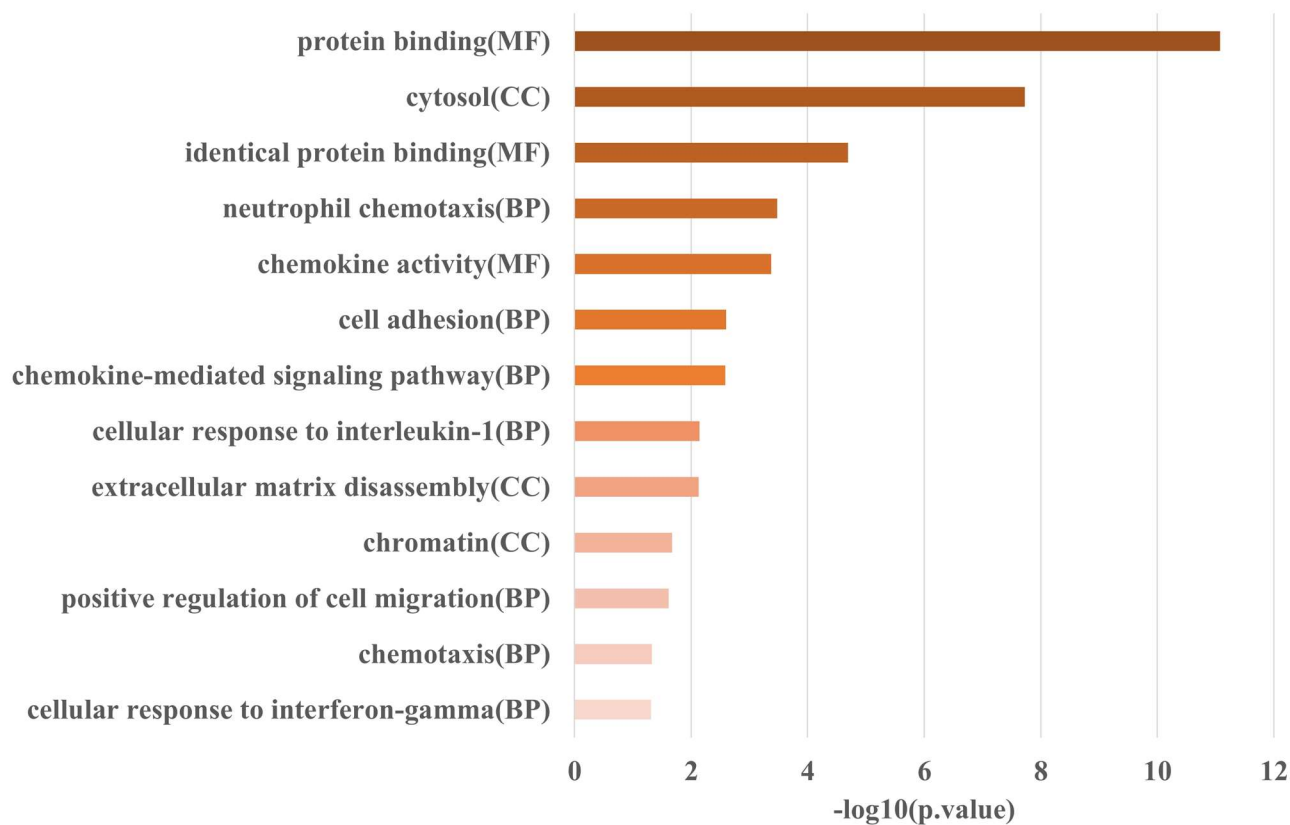

B

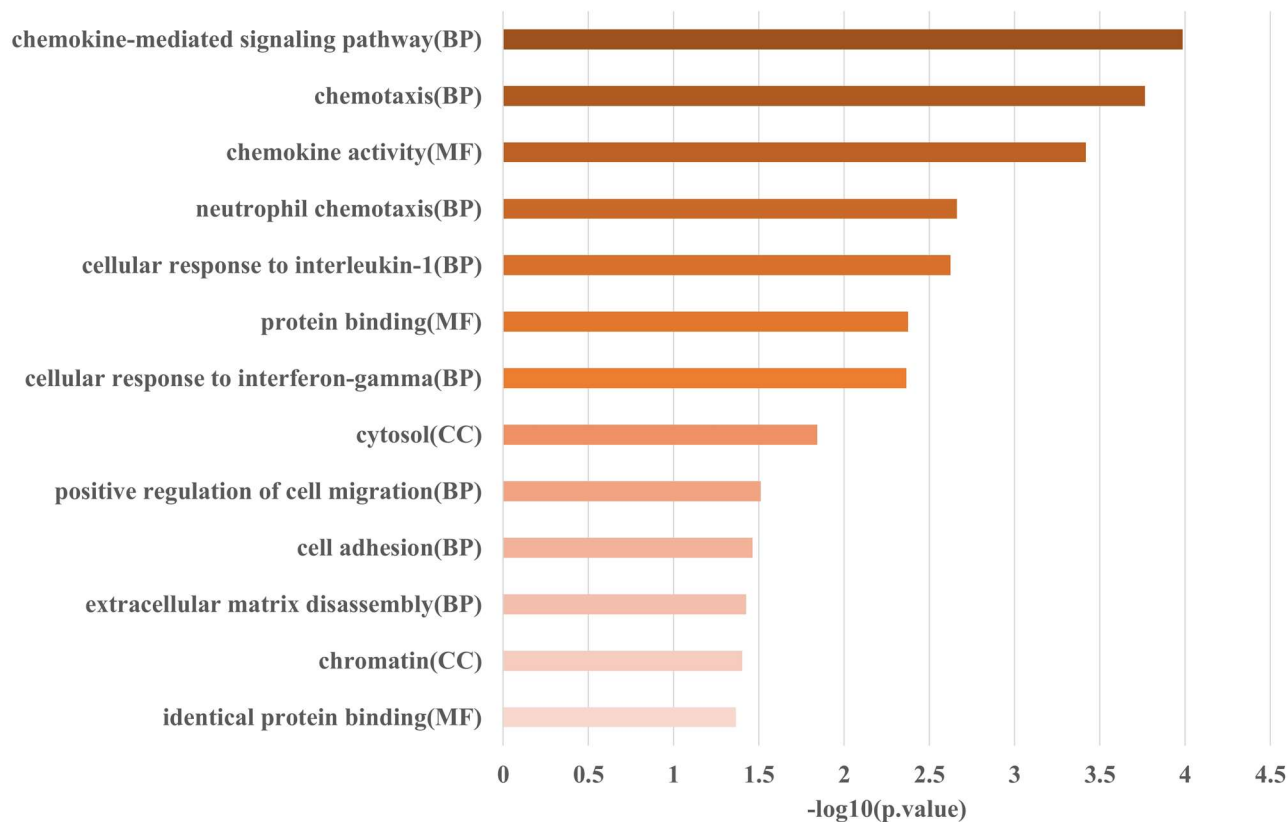

Supplement: Supplementary file 4 — Figure S2 [file CLT2-14-e70012-s002.pdf]

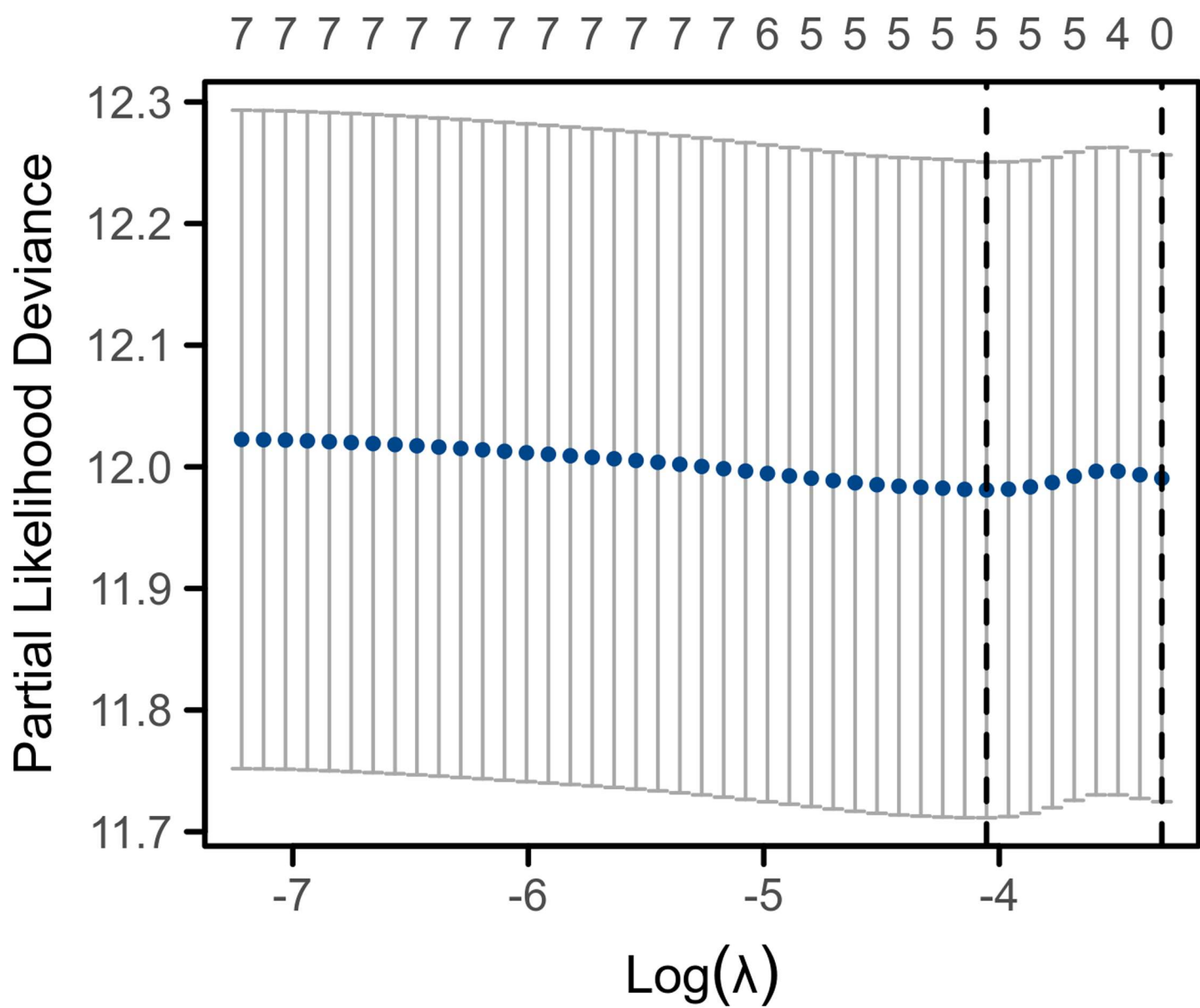

Supplement: Supplementary file 5 — Figure S3 [file CLT2-14-e70012-s004.pdf]

**A**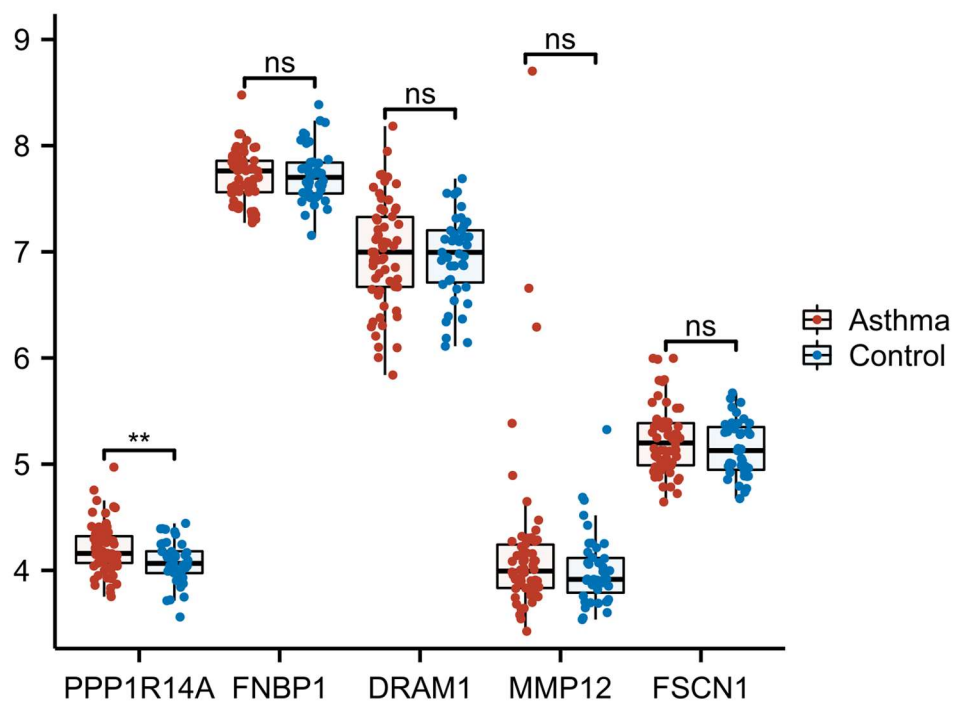**B**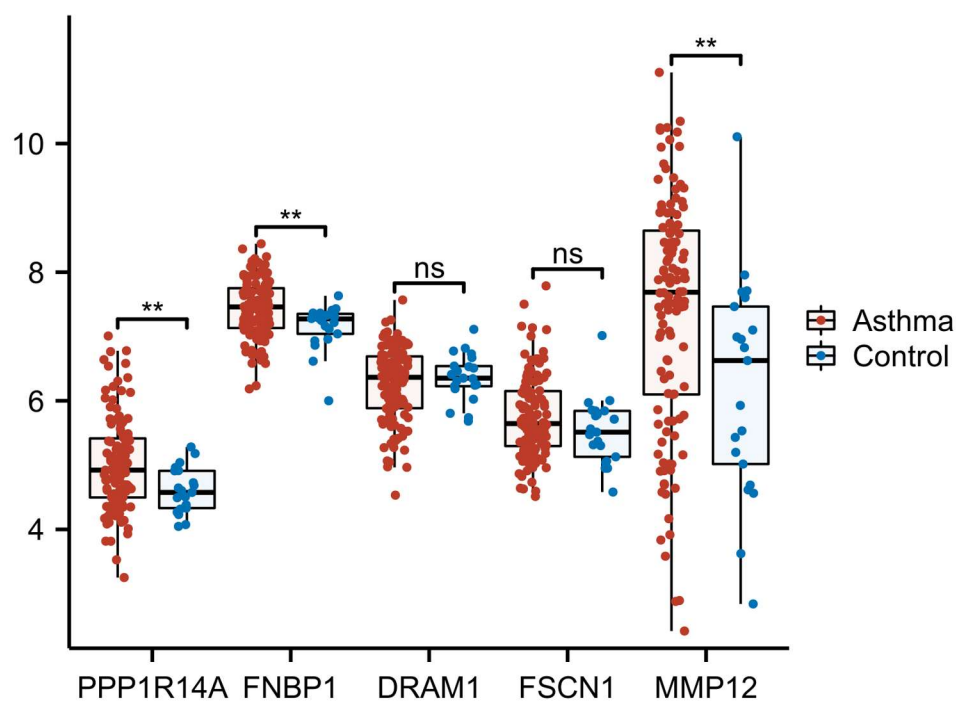**C**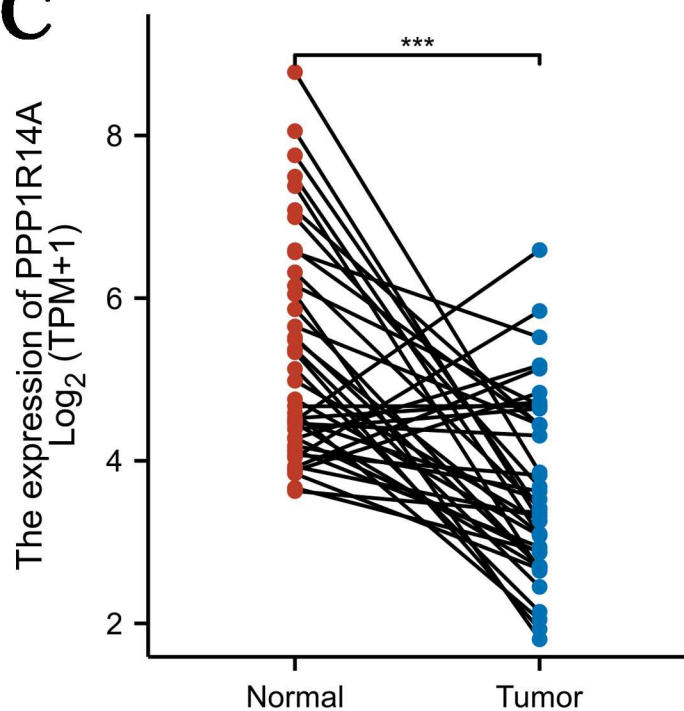**D**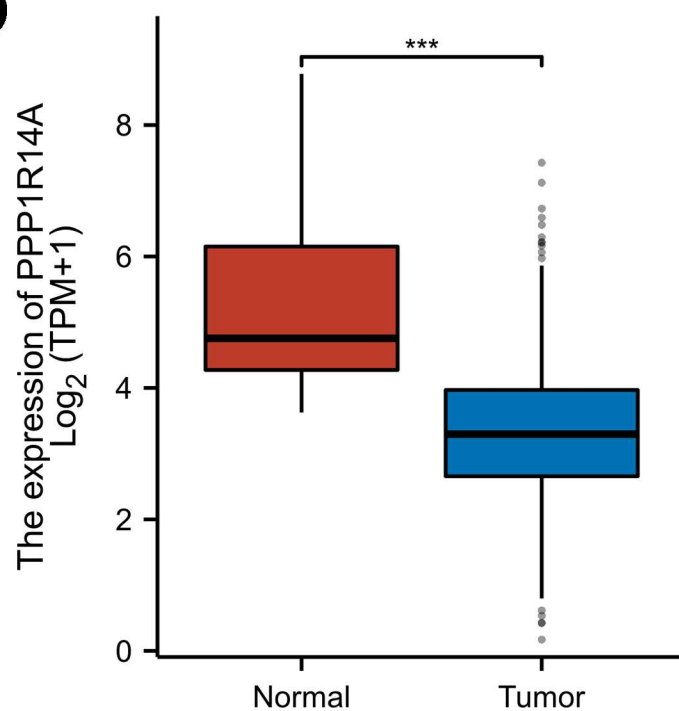

Supplement: Supplementary file 6 — Figure S4 [file CLT2-14-e70012-s003.pdf]
